# CLIC4/Arf6 Pathway: A New Lead in BMPRII Inhibition in Pulmonary Hypertension

**DOI:** 10.1161/CIRCRESAHA.118.313705

**Published:** 2018-10-15

**Authors:** Vahitha B. Abdul-Salam, Giusy Russomanno, Chen Chien-Nien, Abdul S. Mahomed, Luke A. Yates, Martin R. Wilkins, Lan Zhao, Magdalena Gierula, Oliver Dubois, Ute Schaeper, Jens Endruschat, Beata Wojciak-Stothard

**Affiliations:** 1 From the Centre for Pharmacology and Therapeutics (V.B.A.-S., G.R., C.C.-N., A.S.M., M.R.W., L.Z., M.G., O.D., B.W.-S.), Department of Medicine, Imperial College London, United Kingdom; 2 Section of Structural Biology (L.A.Y.), Department of Medicine, Imperial College London, United Kingdom; 3 Silence Therapeutics GmbH, Berlin, Germany (U.S., J.E.).

**Keywords:** chloride channels, endocytosis, endothelial cells, endothelial progenitor cells, hypertension, pulmonary

## Abstract

**Rationale:**

Increased expression of CLIC4 (chloride intracellular channel 4) is a feature of endothelial dysfunction in pulmonary arterial hypertension, but its role in disease pathology is not fully understood.

**Objective:**

To identify CLIC4 effectors and evaluate strategies targeting CLIC4 signaling in pulmonary hypertension.

**Methods and Results:**

Proteomic analysis of CLIC4-interacting proteins in human pulmonary artery endothelial cells identified regulators of endosomal trafficking, including Arf6 (ADP ribosylation factor 6) GTPase activating proteins and clathrin, while CLIC4 overexpression affected protein regulators of vesicular trafficking, lysosomal function, and inflammation. CLIC4 reduced BMPRII (bone morphogenetic protein receptor II) expression and signaling as a result of Arf6-mediated reduction in gyrating clathrin and increased lysosomal targeting of the receptor. BMPRII expression was restored by Arf6 siRNA, Arf inhibitor Sec7 inhibitor H3 (SecinH3), and inhibitors of clathrin-mediated endocytosis but was unaffected by chloride channel inhibitor, indanyloxyacetic acid 94 or Arf1 siRNA. The effects of CLIC4 on NF-κB (nuclear factor-kappa B), HIF (hypoxia-inducible factor), and angiogenic response were prevented by Arf6 siRNA and SecinH3. Sugen/hypoxia mice and monocrotaline rats showed elevated expression of CLIC4, activation of Arf6 and NF-κB, and reduced expression of BMPRII in the lung. These changes were established early during disease development. Lung endothelium–targeted delivery of CLIC4 siRNA or treatment with SecinH3 attenuated the disease, reduced CLIC4/Arf activation, and restored BMPRII expression in the lung. Endothelial colony–forming cells from idiopathic pulmonary hypertensive patients showed upregulation of CLIC4 expression and Arf6 activity, suggesting potential importance of this pathway in the human condition.

**Conclusions:**

Arf6 is a novel effector of CLIC4 and a new therapeutic target in pulmonary hypertension.

## Abstract 2

Supplemental Digital Content is available in the text.

Pulmonary arterial hypertension (PAH) is characterized by increased vasoconstriction, extensive remodeling of small intrapulmonary arteries, and right heart hypertrophy. Endothelial cell injury followed by vascular endothelial and smooth muscle proliferation and endothelial-to-mesenchymal transition contribute to the vascular pathology of PAH.^1^

**Editorial, see p 6**

**Meet the First Author, see p 3**

Inflammation, driven in part by the activation of NF-κB (nuclear factor kappa B), and reduced endothelial BMPRII (bone morphogenetic protein receptor II) expression and/or function are recognized as key factors in endothelial injury and dysfunction that instigate and propagate adverse remodeling of the pulmonary arteries.^2,3^ Expression of BMPRII protein is constitutively regulated by lysosomal degradation in vascular cells,^4^ but regulatory mechanisms have not been fully characterized.

We have recently shown increased expression of CLIC4 (chloride intracellular channel 4) in the lungs of PAH patients and PAH animals.^5^ CLIC4 is a member of CLIC family of proteins (CLIC1-6) involved in the regulation of cell proliferation, apoptosis, angiogenesis, and differentiation.^6,7^ In experimental models of PAH, CLIC4 expression increases in the lung but not in systemic tissues, and deletion of CLIC4 gene in mice prevents development of hypoxia-induced pulmonary hypertension (PH).^5^ CLIC4, like BMPRII localizes preferentially to the pulmonary vascular endothelium and induces the NF-kB-dependent activation of HIF (hypoxia-inducible factor),^5^ but its contribution to BMPRII signaling is not fully understood. While BMPRII knockdown does not alter CLIC4 expression,^5^ CLIC4 affects migratory responses of pulmonary artery smooth muscle cells downstream of BMPRII/BMP (bone morphogenetic protein) signaling.^8^

Targeting CLIC4 with specific small molecule inhibitors has been challenging because of high homology among CLIC proteins. The only existing inhibitor, chloride channel blocker indanyloxyacetic acid 94 used in CLIC research, has a broad spectrum of intracellular targets.^7^ Interestingly, despite their name as intracellular chloride channels, CLIC proteins form poorly selective anion channels and their main function does not depend on chloride conductance.^9^ CLIC4 localizes to endocytotic vesicles^10,11^ and may play a role in the regulation of vesicular trafficking. Vesicular trafficking is controlled by Arf (ADP ribosylation factor) proteins, Ras-related small GTPases that cycle between an active, GTP-bound and an inactive, GDP-bound conformation.^12^ Arf proteins are activated by GEFs (guanine exchange factors) that stimulate exchange of GDP for GTP, thereby switching “on” the GTPase and GAPs (GTPase-activating proteins), which facilitate GTP hydrolysis, thereby switching “off” the GTPase.

Here, we applied an unbiased proteomic screen to identify new targets in CLIC4 signaling pathway and to elucidate CLIC4 contribution to the vascular pathology in PAH. We are first to show a new regulatory pathway in BMPRII signaling involving CLIC4-induced activation of the endosomal trafficking regulator Arf6. We also show that CLIC4 siRNA or Arf inhibitor Sec7 inhibitor H3 (SecinH3) prevent development of PH in 2 preclinical rodent models of PAH.

## Methods

The mass spectrometry proteomics data have been deposited to the ProteomeXchange Consortium via the PRIDE repository with the dataset identifier PXD008709 for CLIC4-interacting proteins and PXD008714 for proteomics of CLIC4 overexpressing human pulmonary artery endothelial cells. Additional data can be obtained from the corresponding author on request. Detailed methods section is available in the Online Data Supplement.

### Endothelial Cell Culture and Treatments

Human pulmonary artery endothelial cells (HPAECs; PromoCell) were cultured in the endothelial growth medium 2 (PromoCell). Overexpression of hemagglutinin-tagged CLIC4 (kind gift from Professor Stuart H. Yuspa, Laboratory of Cancer Biology and Genetics, Centre for Cancer Research, Bethesda) under tetracycline control or AdCLIC4shRNA (Welgen, Inc) was induced by adenoviral gene transfer.^5^ AdGFP (Vector Biolabs) or AdTet-off were used as adenoviral controls (Adcontrol). In some experiments, the cells were exposed to hypoxia (2% O_2_, 5% CO_2_, 92% N_2_) or were incubated with human recombinant TNF-α (tumor necrosis factor α; 10 μg/L; 210-TA-020k, R&D), SecinH3 (10 mg/L; 2849, R&D), NF-κB inhibitor, BAY 117085 (10 μmol/L; Sigma-Aldrich), or a chloride channel inhibitor, indanyloxyacetic acid 94 (100 μmol/L, I117, Sigma-Aldrich) for 24 hours before cell lysis or immunostaining, while human BMP9 (bone morphogenetic protein 9; 10 μg/L; 3209-BP, R&D) or TGF-β (transforming growth factor; 10 μg/L; 240-B-010, R&D) were added to the cells for 1 hour at 37°C.

### Blood-Derived Human Endothelial Cell Culture

Human endothelial colony–forming cells (ECFCs) were derived from peripheral blood samples as previously described.^5^ Venous blood samples were obtained with local ethics committee approval. and informed written consent from healthy volunteers and patients with idiopathic PAH was obtained (clinical information is shown in the Online Table I).

### Proteomic Analysis

HPAECs were left untreated or were infected with Adcontrol or AdCLIC4. Twenty-four hours post-infection, the cells were lysed and subjected to SDS-PAGE (n=4/group). Hemagglutinin-tagged CLIC4 were purified using a hemagglutinin-tagged protein purification kit (3320, MBL Company Ltd) followed by SDS-PAGE separation. Following in-gel digestions, the resultant tryptic peptides from each experiment were separated on a nano-liquid chromatography–tandem mass spectrometry for identification and quantitation. For overexpression analysis, differentially expressed proteins were identified on the basis of at least 1.5-fold difference in abundance in AdCLIC4 versus AdControl with *P* value <0.05. Ingenuity Pathway Analysis (Qiagen) was used for mapping the identified proteins to known biological pathways. For CLIC4-interacting protein analysis, only the proteins that were unique to the hemagglutinin-tag purification fraction were listed.

### Cell Transfection

Electroporation of plasmid DNA with BMPRII-GFP (RG208673; Origene), pRK5F-PPM1A (protein phosphatase 1A plasmid DNA; kind gift of Professor Xin-Hua Feng, Baylor College of Medicine, TX), pmaxGFP (Lonza), YFP-GGA1 (yellow fluorescent protein–labelled golgi-associated, gamma adaptin ear-containing, ARF-binding protein 1)^13^ or mCherry clathrin^14^ (kind gift of Professor James Keen, Thomas Jefferson University, Philadelphia), Silencer Select human siRNAs: negative control siRNA No. 1 (4390843; Ambion), Arf1 siRNA (4390824, ID: s1552; Ambion), Arf6 siRNA (4390824, ID: s1565; Ambion) were conducted using the Amaxa Basic Nucleofector Kit (VPI-1001, Lonza). Cell responses were studied 48 hours post-transfection. In some experiments, silencing RNAs were transfected into cells using Lipofectamine RNAiMAX (13778075; ThermoFisher Scientific) according to the manufacturer’s protocol.

### Immunostaining and Western Blot Analysis

CLIC4, p65 NF-κB, BMPRII, Smad1, Smad5 and p-Smad1,5, Smad3 and p-Smad3, Arf1, Arf6, LAMP-1 (lysosomal-associated membrane protein 1), β-actin, and filamentous-actin were studied by Western blotting and immunofluorescence in cells, cell lysates, or tissue sections, as appropriate.

### Microscale Thermophoresis

Microscale thermophoresis was performed to study the interaction between enhanced green fluorescent protein–labelled G-protein–coupled receptor kinase-interacting protein 1 (GIT1; 15223; Adgene) in human embryonic kidney 293 cells lysate and a serial dilution of purified CLIC4 (ab104744; Abcam) using Monolith NT.115 instrument according to the manufacturer’s protocol (NanoTemper Technologies, Germany). The assay was performed with constant concentration of ECFP-labeled GIT1 at 20 nmol/L and varying concentrations of CLIC4.

### Arf6 Activity Assay

Arf6 and Arf1 activity (GTP-binding) assays were performed according to the manufacturer’s protocol (BK033-S and BK032-S, respectively; Cytoskeleton, Inc).

### Proximity Ligation Assay

Intracellular distribution and spatial proximity of CLIC4 and BMPRII in HPAECS were studied with Duolink in situ proximity ligation assay according to manufacturer’s instructions (Olink Biosciences; Sigma-Aldrich). Samples were mounted with Duolink mounting media, and cell images were taken under a fluorescent confocal microscope. The number of fluorescent puncta indicative of the close proximity of the 2 molecules was scored by measuring the intensity of fluorescence in each cell with Image J.

### NF-κB Luciferase Reporter Assay and Nuclear Translocation of p65NF-κB

NF-κB luciferase reporter assay was performed as previously described.^5^ Nuclear translocation of p65 NF-κB was studied with Image J. The white pixel area, marking nuclear NF-κB, was used to quantitate p65 NF-κB translocation in cells and tissues.

### HIF Activation and Tube Formation Assay

HIF-1α stabilization and tube formation in matrigel were studied as previously described.^5^

### Quantification of Gyrating (G-) Clathrin

HPAECs transfected with plasmid encoding YFP-GGA1 were infected with Adcontrol or AdCLIC4 and incubated with, or without, SecinH3 (10 mg/L), as appropriate. Twenty-four hours later, live cells were recorded under the confocal laser scanning fluorescence microscope (Zeiss LSM-780 at the facility for imaging by light microscopy) to collect frame image stacks. Quantification of gyrating clathrin (G-clathrin) was performed as previously described.^15^

### Lysosome Markers and Lysosome Acidification

Lysosome content was evaluated by measuring fluorescence intensity of confluent cells immunostained for lysosome marker protein LAMP1, while lysosome acidification was evaluated with pHrodo Red, succinimidyl ester (pHrodo Red, SE, P36600; Thermofisher Scientific), according to the manufacturer’s protocol. pHrodo Red dye conjugates are nonfluorescent outside the cell, but fluoresce brightly red in phagosomes. Three confocal images comprising ≈200 cells/coverslip on 3 coverslips per treatment in 3 separate experiments, were analyzed.

### Clathrin-Mediated Endocytosis

Clathrin-mediated endocytosis was evaluated by measuring the uptake of transferrin from Human Serum, Alexa Fluor 488 Conjugate (T13342, Thermofisher Scientific). Clathrin-independent endocytosis was measured by intake of mouse monoclonal antibodies directed toward MHC-I (major histocompatibility complex I; clone w6/32, Biolegend) or control mouse monoclonal anti-cytokeratin 14 antibody (ab7800; Abcam; antibodies directed against intracellular target), followed by removal of the unbound antibody by low pH acid wash, fixation, and staining with secondary fluorescein isothiocyanate–labeled goat anti-mouse antibody (115-095; Jackson Laboratories).^16^ In some experiments, control (Adcontrol) and CLIC4-overexpressing (AdCLIC4) cells were treated with 20 mg/L protein synthesis inhibitor cycloheximide (CAS 66-81-9; Santa Cruz Biotechnology), with or without the endocytosis inhibitor Pitsop2 (20 μmol/L; ab120687; Abcam)^16^ or Pitstop2-negative control (20 μmol/L; ab120688; Abcam,) for 2 hours. Pitsop2-negative control is chemically related to Pitstop2 but does not block receptor-mediated endocytosis. BMPRII expression was analyzed by Western blotting.

### Plasma Membrane Anion Permeability

Cell plasma membrane anion permeability was assessed using the Chloride Channel Assay Kit from Abcam (ab176767), which measures passive cellular iodide uptake, according to the manufacturer’s protocol.

### Animal Experiments

All experiments were conducted in accordance to the UK Home Office Animals (Scientific Procedures) Act 1986 (London, United Kingdom). Detailed description of experimental procedures can be found the Online Data Supplement. All animals were randomly allocated to groups, and all personnel involved in data collection and analysis (hemodynamics and histopathologic measurements) were blinded to the treatment status of each animal. Only weight- and age-matched males were included for experimentation as, in contrast to the human clinical studies, most animal studies have shown that female sex and estrogen supplementation have a protective effect against PAH.^17^

Three series of experiments were conducted. In the first 2 series of experiments, a liposomal formulation DACC/CLIC4siRNA or SecinH3 were delivered to Sugen/hypoxia mice from the start of disease development. In the third series of experiments, SecinH3 was delivered to monocrotaline rats 8 days post monocrotaline injection.

In Sugen/hypoxia mouse model of PAH, adult male C57/Bl6 mice (20 g, 8/group) were injected subcutaneously with Sugen (SU5416; 20 mg/kg; Tocris Bioscience), suspended in 0.5% (w/v) carboxymethylcellulose sodium, 0.9% (w/v) sodium chloride, 0.4% (v/v) polysorbate 80, 0.9% (v/v) benzyl alcohol in deionized water once per week. Control mice received only vehicle. The animals were exposed to chronic normobaric hypoxia (10% O_2_) in a ventilated chamber for 21 days. DACC/CLIC4siRNA or DACC control nontargeting siRNA were delivered via intravenous injection (2.8 mg/kg body weight) twice a week. Single injection of fluorescently labeled DACC/siRNA-Cy3 (2.8 mg/kg body weight) in healthy mice was used to follow distribution of siRNA in lungs, heart, liver, and kidney 4 hours and 24 hours post-injection. Localization of the fluorescent mimic to the endothelium was confirmed by co-immunostaining for VWF (von Willebrand Factor). Preparation and characterization of siRNA lipoplexes was performed as described.^18^

In the second group, Sugen/hypoxia mice (n=8/group) were injected every other day intraperitoneally either with vehicle (20% DMSO and 10% Tween-20) or SecinH3 (100 μL of 5 mmol/L, 2849, Tocris Bioscience) dissolved in the vehicle. The normoxic control group received vehicle only. Following 21-day hypoxic exposure, the animals were removed from the hypoxic chamber individually and anesthetized by intraperitoneal injection of Ketamine/Dormitor (75 mg/kg+1 mg/kg).

In the third group, male Sprague-Dawley rats (190–200 g, n=6/group) were injected subcutaneously with a single dose of monocrotaline (60 mg/kg body weight). Eight days after monocrotaline injection, 6 rats were injected intraperitoneally every other day with vehicle (20% DMSO and 10% Tween-20), while the other group (n=6) was injected with SecinH3 (2.5 mg/kg body weight) dissolved in vehicle, for a total of 14 days. Control rats (no monocrotaline ) were injected with vehicle intraperitoneally every other day. After 21 days of the study, rats were anesthetized with fentanyl/fluanisone (Hypnorm, VetaPharma) using intramuscular injection at 1 mL/kg body weight; followed by intraperitoneal injection with 0.8 mL/kg body weight of midazolam (Hypnovel, Roche). Changes in CLIC4, Arf6, and BMPRII expression were evaluated at 3, 7, 14, and 21 days post-monocrotaline injection.

Development of PAH was assessed by measuring right ventricular systolic pressure (RVSP) in mice and mean pulmonary artery pressure in rats and right ventricular hypertrophy (RVH) denoted by the right ventricle to left ventricle/septum ratio. In mice, pulmonary vascular remodeling (muscularization of small intrapulmonary arteries) was determined by counting all muscularized (showing thickened α-SMA-positive media) vessels with a diameter smaller than 50 μm in each lung section and expressed as a percent of all (muscularized+nonmuscularized) vessels. In rats, vascular remodeling was determined as the proportion of peripheral vessels (<100 μm in diameter) with double elastic lamina visualized with elastic van Gieson staining (>75% of the circumference as fully muscularized, 25% to 75% as partly muscularized) to total vessels counted.

### Quantitative Reverse Transcriptase Polymerase Chain Reaction

For target mRNA knock down analyses, tissues were dissected immediately after killing of the mice and instantly snap-frozen in liquid nitrogen. Twenty-five to 100 ng total RNA was used for quantitative reverse transcriptase polymerase chain reaction with the amplicon sets listed in Online Data Supplement. Data were calculated by using the comparative Ct method.

### Statistical Analysis

All experiments were performed at least in triplicate. Data are presented as means±SEM. Normality of data distribution was assessed with Shapiro-Wilk test in GraphPad Prism 7.03. Comparisons between 2 groups were made with Student *t* test or Mann-Whitney’s *U* test, whereas ≥3 groups were compared by use of ANOVA with Tukey’s post hoc test or Kruskal-Wallis with Dunn’s post hoc test, as appropriate. Statistical significance was accepted at *P*<0.05.

## Results

### CLIC4 Associates With Protein Regulators of Nf-κB Signaling and Vesicular Trafficking

To identify potential therapeutic targets in the CLIC4 signaling pathway, we performed a proteomic screen of CLIC4-interacting proteins, using C-terminal hemagglutinin tag as bait. The identified set of proteins included p65NF-kB and key regulators of the endocytotic pathway such as Arf6 GAP proteins (GIT1 and GIT2), clathrin heavy chain, dynein, actin crosslinking proteins, and tubulin (Table; Online Table II). The association of CLIC4 with clathrin heavy chain and GIT1 was verified by co-immunoprecipitation followed by Western blotting (Online Figure I). Further, direct binding between CLIC4 and GIT1 was confirmed using microscale thermophoresis. Microscale thermophoresis permits a robust analysis of protein-protein interactions in cell lysates and determination of their binding affinity (denoted by *K*_*d*_ values), thereby quantitating the interaction in a close-to-native context.^19^ The estimated *K*_*d*_ for the interaction between CLIC4 and GIT1 was 1.8±0.3 μmol/L (Online Figure II), while no binding was observed in BSA control.

**Table.**
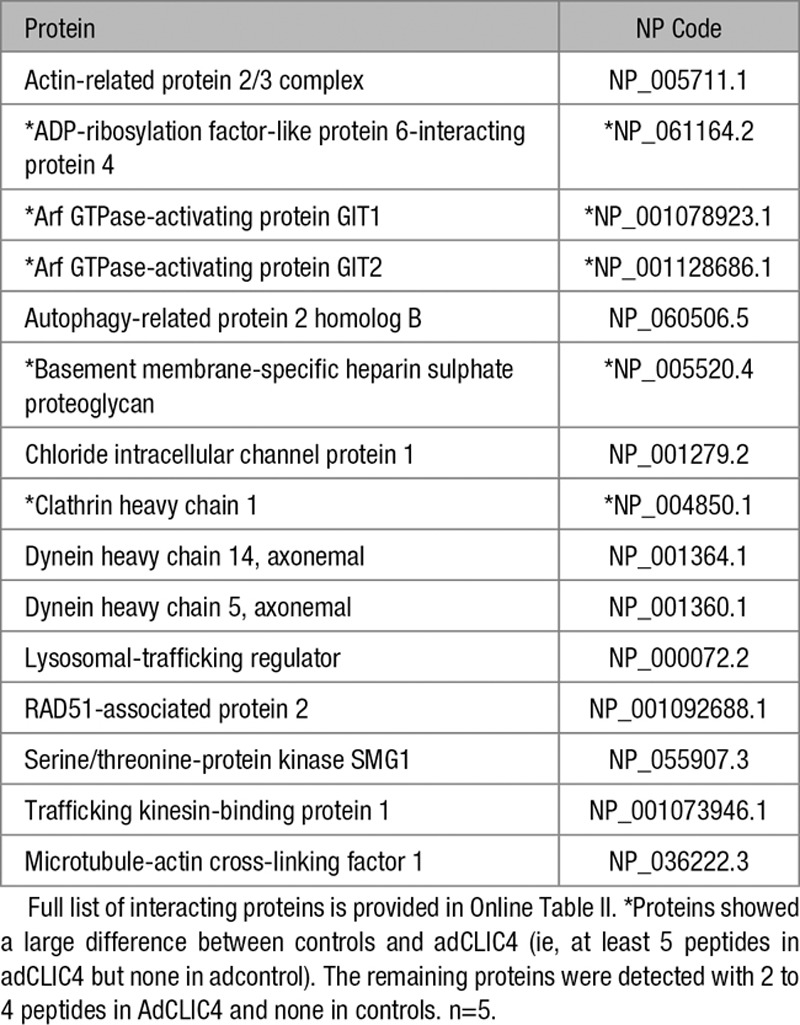
CLIC4-Interacting Proteins Involved in the Regulation of Endocytotic Trafficking

To gain a better understanding of CLIC4 function, we performed a label-free GeLCMS proteomic analysis^20^ of CLIC4-overexpressing HPAECs. Differentially expressed proteins showed pathway association with endocytotic trafficking, lysosomal degradation, NF-κB, and TNF-α signaling (Online Table III). Standardized CLIC4 expression levels and changes in the expression of selected proteins were confirmed by Western blotting (Online Figure III).

### CLIC4 Reduces BMPRII Expression and Signaling and Enhances Inflammatory Responses in HPAECs

**Figure 1.**
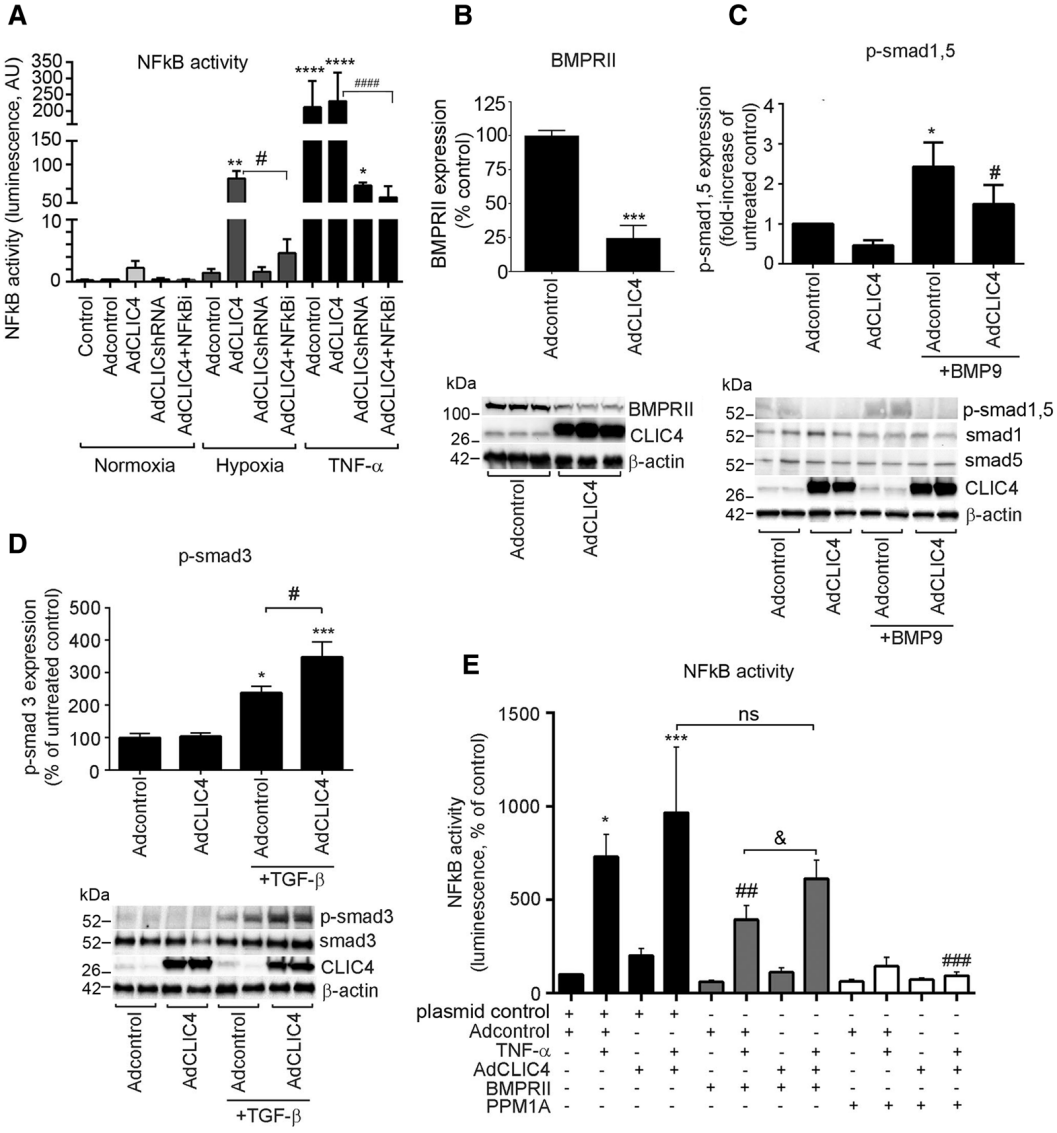
**CLIC4 increases NF-κB activity and reduces BMPRII signaling in HPAECS.** **A**, NF-κB activity in HPAECs overexpressing CLIC4 or CLIC4shRNA in normoxia, hypoxia, and in cells treated with TNF-α (10 μg/L, 24 h) or NF-κB inhibitor, BAY 117085 (10 μmol/L, 24 h); luciferase reporter assay. (**B**) BMPRII protein levels, (**C**) Smad1/5 phosphorylation, and (**D**) Smad3 phosphorylation in HPAECs infected with Adcontrol or AdCLIC4. In (**C**), the cells were treated with BMP9 (10 μg/L, 1 h) and in (**D**) with TGF-β (10 μg/L, 1 h). Representative Western blots are shown underneath the graphs. **E**, The effect of BMPRII and PPM1A overexpression on CLIC4-induced activation of NF-κB in cells treated, as indicated. *P<0.05; **P<0.01, ***P<0.001, ****P<0.0001, comparisons with Adcontrol; #*P*<0.05; ##*P*<0.01; ###*P*<0.001, comparisons with CLIC4+TNF-α or as indicated. Data are presented as mean±SEM; n=4–8. Student *t* test or 1-way ANOVA with Tukey’s post-test, as appropriate. BMP9 indicates bone morphogenetic protein 9; BMPRII, bone morphogenetic protein receptor II; CLIC4, chloride intracellular channel 4; HPAECs, human pulmonary artery endothelial cells; PPM1A, protein phosphatase 1A; NF-κB, nuclear factor kappa B; and TNF-α, tumor necrosis factor α.

Reduced BMPRII signaling and activation of the inflammatory transcription factor NF-κB play a central role in the pathogenesis of PAH. CLIC4 overexpression markedly enhanced hypoxia-induced activation of NF-κB but had little additional effect on TNF-α-stimulated cells, possibly because the response was already maximal under these conditions. CLIC4shRNA and the NF-κB inhibitor, BAY 117085, reduced NF-κB activity in all experimental conditions (Figure 1A). NF-κB activation was accompanied by increased nuclear localization of p65NF-κB (Online Figure IV).

CLIC4 markedly reduced protein levels of BMPRII (Figure 1B; Online Figure V) and reduced phosphorylation of Smad1 and Smad5 in response to BMP9 stimulation (Figure 1C). CLIC4 also augmented TGF-β-induced phosphorylation of Smad3 (Figure 1D), possibly as a result of contemporary inhibition of BMPRII signaling. Overexpression of BMPRII had no effect on CLIC4-induced NF-κB activation, but overexpression of serine phosphatase PPM1A, a protein thought to compete with CLIC4 for NF-κB binding,^6^ had an inhibitory effect in all experimental conditions (Figure 1E; Online Figure VI)

To investigate whether the effects of CLIC4 on NF-κB activity can be related to changes in anion flux, we measured plasma membrane anion permeability in CLIC4-overexpressing normoxic and hypoxic HPAECs. CLIC4 induced a modest (≈2-fold) increase in plasma membrane anion permeability in both normoxic and hypoxic cells (Online Figure VII). In comparison, CLIC4 overexpression elicited a differential NF-κB response, such that it induced a much more pronounced (≈40-fold) increase in NF-κB activity in hypoxic versus normoxic cells (Figure 1A). CLIC4-induced decrease in BMPRII expression was unaffected by chloride channel blocker indanyloxyacetic acid 94 (Online Figure VIII).

### CLIC4 Redirects Endosomal Cargo to Lysosomal Degradation

**Figure 2.**
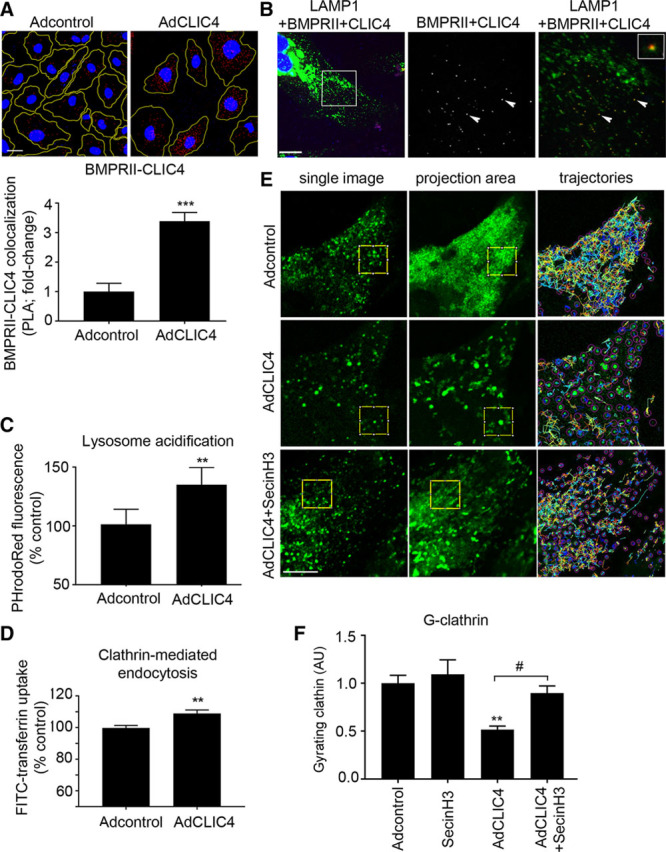
**The effect of CLIC4 on intracellular localization of BMPRII, lysosomal function, and clathrin-mediated vesicular trafficking.** Confocal images and graph in (**A**) show increased colocalization of BMPRII and CLIC4 in CLIC4-overexpressing cells; proximity ligation assay (PLA). **B**, Colocalization of CLIC4 and BMPRII in LAMP1-positive vesicles, PLA. Boxed area in the **left** image is magnified in the middle and right images, which show colocalization of BMPRII with CLIC4 (white pixels, middle image) or colocalization of CLIC4 and BMPRII (red) with LAMP1 (green) in the **right** image. Arrowheads indicate colocalization points with 1 enlarged colocalization point in the inset in the **right** image. **C**, Endosomal/lysosomal acidification; **D**, clathrin-mediated uptake of Alexa488-transferrin. **E**, Single confocal images of YFP-GGA1 (yellow fluorescent protein–labelled golgi-associated, gamma adaptin ear-containing, ARF-binding protein 1) vesicles (**left**), sum projection images from image stacks (**middle**), and trajectories of YFP-GGA1 vesicles (**right**) in cells treated, as indicated; **F**, levels of gyrating clathrin (G-clathrin) in control and CLIC4-overexpressing HPAECs, with or without Sec7 inhibitor H3 (SecinH3; 10 mg/L), as indicated. In all images Bar=10 μm. **P<0.01, ***P<0.001, Student *t* test, or 1-way ANOVA with Tukey’s post-test, as appropriate; n=4–6. Data are presented as mean±SEM. BMPRII indicates bone morphogenetic protein receptor II; CLIC4, chloride intracellular channel 4; HPAECs, human pulmonary artery endothelial cells; and LAMP1, lysosomal-associated membrane protein 1.

The involvement of CLIC4 in the regulation of endocytotic pathway and lysosomal trafficking documented by proteomic analysis was verified in a number of functional assays in cultured HPAECs. Proximity ligation assay showed close colocalization of BMPRII and CLIC4 in LAMP1-positive late endosomes/lysosomes, which was increased upon CLIC4 overexpression (Figure 2A through 2C). CLIC4 increased endosomal/lysosomal acidification and increased the levels of LAMP1-positive lysosomes (Figure 2D; Online Figure IX).

Proteomic analysis identified clathrin as one of the CLIC4-interacting proteins. Clathrin regulates internalization and trafficking of numerous membrane receptors,^21^ including TfR (transferrin receptor) and BMPRII. In HPAECs, CLIC4 enhanced clathrin-mediated internalization of fluorescently labeled transferrin, without affecting clathrin-independent^16^ internalization of MHC-I (Figure 2E; Online Figure IX). To further validate the role of endocytotic receptor internalization in CLIC4-induced changes in BMPRII expression, CLIC4-overexpressing cells were treated with endocytosis inhibitor Pitstop2,^16,22^ together with protein synthesis inhibitor, cycloheximide. BMPRII is constitutively internalized in HPAECs, and treatment with protein synthesis inhibitor cycloheximide for 2 hours results in complete depletion of the intracellular pool of BMPRII.^23^ Pitstop2 restored BMPRII expression in CLIC4-overexpressing cells (Online Figure X).

Recent findings show that a submembrane population of fast-moving clathrin vesicles called gyrating clathrin is responsible for directing the cargo of sorting endosomes directly to the cell surface and that changes in clathrin light or heavy chains or destabilization of Arf6 GDP-GTP cycle compromise its function.^15^ We measured the levels of G-clathrin by analyzing motility of YFP-GGA1-labeled intracellular vesicles in the presence of the fungal metabolite, brefeldin. G-clathrin structures are brefeldin-resistant and colocalize (≈97%) with expressed YFP-GGA1, a guanine nucleoside exchange factor for Arf6 and therefore can be analyzed separately from other endosomal structures and Golgi vesicles.^24^ Time lapse recording of the highly motile YFP-GGA1-positive vesicles in live cells showed that CLIC4 overexpression reduced the pool of gyrating clathrin in HPAECs, while treatment of cells with Arf GEF inhibitor, SecinH3, prevented this effect (Figure 2E and 2F). SecinH3 inhibits the activity of Arf GEFs, cytohesins 1 to 3, by binding to their Sec7 domain, without formation of a complex with Arf.^25,26^

To summarize, CLIC4 augmented internalization of BMPRII into the endosomal and lysosomal compartments and inhibited G-clathrin, critical for the recycling of the endosomal cargo back to the plasma membrane.

### CLIC4 Effects Are Mediated by Arf6

**Figure 3.**
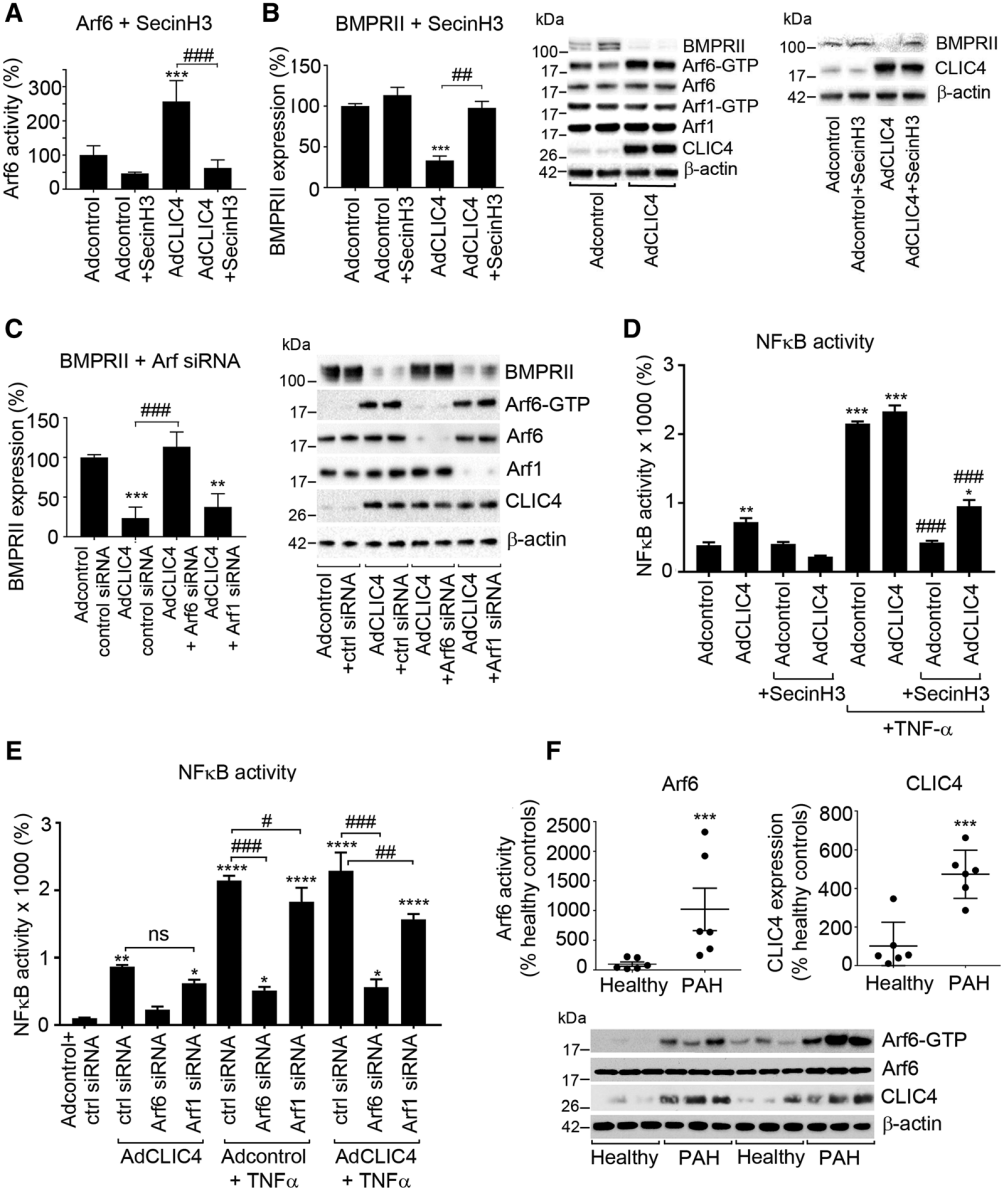
**Arf6 mediates the effects of CLIC4.** **A**, Arf6 activity; **B**, BMPRII expression in control HPAECs (Adcontrol), HPAECs overexpressing CLIC4 (AdCLIC4), with or without Sec7 inhibitor H3 (SecinH3; 10 mg/L, 24 h), as indicated. Representative Western blots are shown on the **right**. Graph in (**C**) and corresponding representative Western blots show the effect of control siRNA (ctrl siRNA), Arf6 siRNA, and Arf1 siRNA on BMPRII levels in Adcontrol- and AdCLIC4-overexpressing cells. **D**, The effect of SecinH3 on TNF-α-induced activation of NF-κB in control and CLIC4-overexpressing HPAECs; **E**, The effect of Arf6 siRNA and Arf1 siRNA on TNF-α-induced activation of NF-κB in cells treated, as indicated. **F**, Arf6 activity and CLIC4 expression in ECFCs from IPAH patients, n=6. *P<0.05, **P<0.01, ***P<0.001, comparisons with Adcontrol; #*P*<0.05; ##*P*<0.01; ###*P*<0.001, comparisons with AdCLIC4+TNF-α or as indicated. Data are presented as mean±SEM; n=4–6. Student *t* test or 1-way ANOVA with Tukey post hoc test, except for (**F**), where data were analyzed with Mann-Whitney *U* test. Arf6 indicates ADP ribosylation factor 6; BMPRII, bone morphogenetic protein receptor II; CLIC4, chloride intracellular channel 4; HPAECs, human pulmonary artery endothelial cells; NF-κB, nuclear factor kappa B; and TNF-α, tumor necrosis factor α.

We found that CLIC4 interacts with the Arf6 GAPs, GIT1 and GIT2, the key regulators of receptor trafficking at the plasma membrane.^12^ While GIT1 and GIT2 co-localize with and specifically activate Arf6,^27^ they may show some catalytic activity toward Arf1.^28^ Arf1 and Arf6 differ in their function and subcellular localization: Arf1 localizes to and acts within Golgi, whereas Arf6 localizes to and acts at the cell periphery.^29^ We measured Arf6 and Arf1 activity and expression in CLIC4-overexpressing cells and observed that CLIC4 increased Arf6 activity 3-fold without affecting the activity or expression of Arf1 (Figure 3A through 3C). No cross-reactivity between anti-Arf1 and anti-Arf6 antibodies was detected (Online Figure XI). Treatment of CLIC4-overexpressing cells with SecinH3 and Arf6 siRNA restored BMPRII expression, while Arf1 siRNA had no effect, indicating that CLIC4-mediated regulation of BMPRII expression is Arf6-specific (Figure 3A through 3C). SecinH3 and Arf6siRNA prevented CLIC4-induced activation of NF-κB, HIF, and tube formation in vitro (Figure 3D and 3E; Online Figure XII). Arf1siRNA did not affect CLIC4-induced responses, though it had some inhibitory effect on TNF-α-induced NF-κB activation and endothelial tube formation (Figure 3E; Online Figure XII), likely to result from its key role in the regulation of Golgi dynamics. Collectively, these results confirm the role of Arf6 as a key mediator of CLIC4-induced responses.

Importantly, ECFCs from idiopathic PAH patients showed 10-fold increase in Arf6 activity and 5-fold increase in CLIC4 expression, compared with the cells from healthy volunteers (Figure 3F), suggesting a potential role for this pathway in human disease.

### Lung Endothelium-Targeted Liposomal Delivery of CLIC4siRNA Attenuates Development of PH, Reduces NF-κB Activity, and Restores Lung Expression of BMPRII in Sugen/Hypoxia Mice

**Figure 4.**
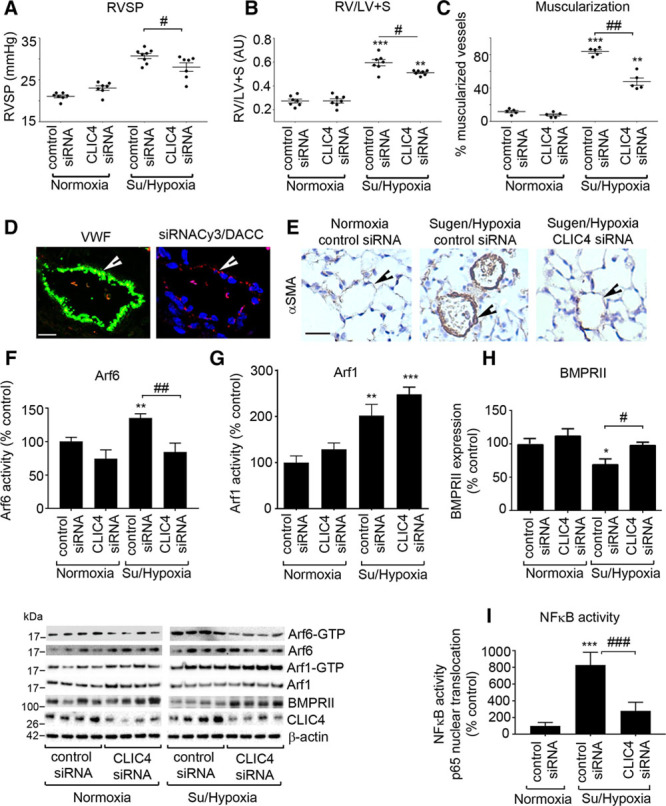
**Effects of CLIC4siRNA on development of pulmonary hypertension, Arf activation, BMPRII expression, and NF-κB activity in Sugen/hypoxia mice.** (**A**) RVSP; (**B**) RV/LV+S; and (**C**) percentage of muscularized vessels in lungs of control mice and Sugen/hypoxia mice treated with nontargeting siRNA (control siRNA) or CLIC4 siRNA/DACC lipoplex, as indicated. **D**, Confocal images showing endothelial localization of nontargeting fluorescent siRNA delivered by DACC delivery vehicle (siRNACy3/DACC); Bar=10 μm. **E**, αSMA staining in mouse lung sections. Arrowheads point to small intrapulmonary vessels. Bar=25 μm. **F–H**, Graphs and corresponding representative Western blots show Arf6 and Arf1 activity, CLIC4, and BMPRII expression in lungs of the untreated and CLIC4siRNA-treated mice, as indicated. **I**, NF-κB activity in mice treated, as indicated. *P<0.05, **P<0.01, ***P<0.001, comparisons with controls; #*P*<0.05, ###*P*<0.001 comparisons with control siRNA Sugen/hypoxia group or as indicated. Data are presented as mean±SEM; n=7–8. One-way ANOVA with Tukey post hoc test. Arf6 indicates ADP ribosylation factor 6; BMPRII, bone morphogenetic protein receptor II; CLIC4, chloride intracellular channel 4; NF-κB, nuclear factor kappa B; LV, left ventricle; RV, right ventricular; RVSP, right ventricular systolic pressure; and VWF, von Willebrand Factor.

To assess the functional role of CLIC4/Arf6 in experimental models of PAH, we used DACC siRNA delivery system to target CLIC4siRNA to the lung endothelium.^18^ The analysis of the tissue distribution of siRNA-Cy3-labeled DACC formulation confirmed that DACC directs the siRNA predominantly to the lung endothelium (Figure 4D; Online Figure XIII), consistent with previously published data.^18^ Treatment with DACC/CLIC4siRNA formulation significantly reduced CLIC4 mRNA and protein levels in the lungs of these animals (Online Figure XIV).

Sugen/hypoxia induced a significant increase in RVSP, RVH, and intrapulmonary small vessel muscularization marked by prominent α-SMA staining seen in normally nonmuscularized, precapillary arterioles (Figure 4A through 4C and 4E; Online Figure XV). These changes were accompanied by a marked activation of Arf6 and Arf1, increase in CLIC4 expression, and reduction in protein expression of BMPRII (Figure 4F through 4I). DACC/CLIC4 siRNA markedly reduced RVSP (*P*<0.05), RVH (*P*<0.001), and vascular muscularization (*P*<0.001), while control, nontargeting siRNA had no significant effect (Figure 4A through 4C and 4E). Consistent with the effects observed in cultured cells, the in vivo application of CLIC4siRNA markedly reduced Arf6 activity, increased BMPRII expression, and reduced NF-κB activation in the lung but had no significant impact on Arf1 activity (Figure 4F through 4I; Online Figure XIV). Protein levels of Arf6 and BMPRII mRNA levels were not affected by the treatment (Online Figure XIV and data not shown).

### SecinH3 Prevents Development of PH in Sugen/Hypoxia in Mice and Attenuates Symptoms of Disease in Monocrotaline Rats

**Figure 5.**
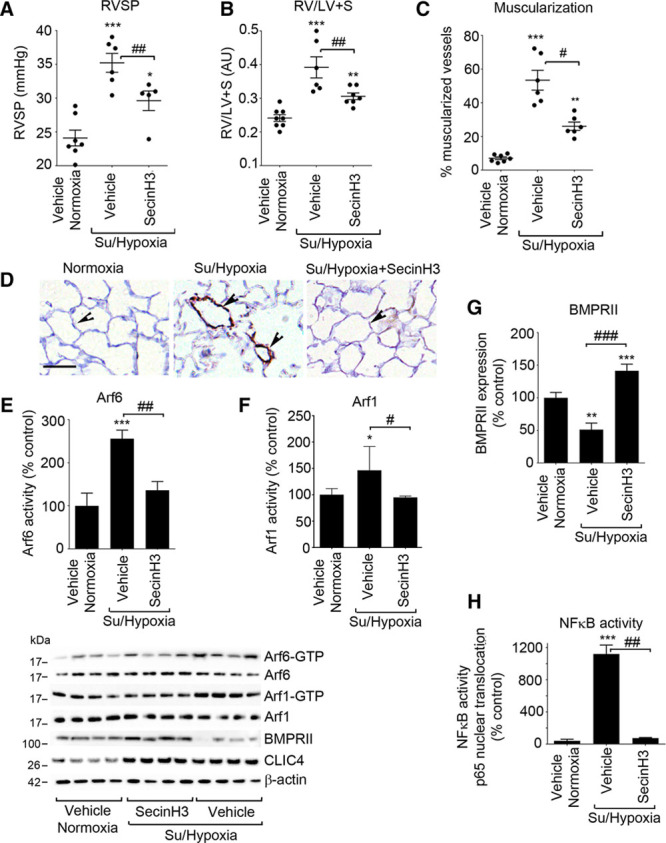
**Effects of Sec7 inhibitor H3 (SecinH3) on development of pulmonary hypertension, Arf activation, BMPRII expression, and NF-κB activity in Sugen/hypoxia mice.** **A**, RVSP; (**B**) RV/LV+S, and (**C**) percentage of muscularized vessels in lungs of control mice and Sugen/hypoxia mice treated with SecinH3, as indicated. **D**, αSMA staining in mouse lung sections. Arrowheads point to small intrapulmonary vessels. Bar=25 μm. (**E**) Arf6 activity, (**F**) Arf1 activity, and (**G**) BMPRII expression in the lungs of untreated and SecinH3-treated mice, as indicated. Representative Western blots are shown below the graphs. **H**, NF-κB activity in mice. *P<0.05, **P<0.01, ***P<0.001, comparisons with controls; #*P*<0.05, ##*P*<0.01, ###*P*<0.001 comparisons, as indicated. Data are presented as mean±SEM; n=8. One-way ANOVA with Tukey post hoc test. Arf6 indicates ADP ribosylation factor 6; BMPRII, bone morphogenetic protein receptor II; NF-κB, nuclear factor kappa B; LV,left ventricle; RV, right ventricle; and RVSP, right ventricular systolic pressure.

Intraperitoneal delivery of SecinH3 reduced RVSP, RVH, and reduced pulmonary muscularization in Sugen/hypoxia mice (Figure 5A through 5D; Online Figure XVI). This effect was associated with increased BMPRII expression and reduced activity of Arf6 and NF-κB in the lung (Figure 5E through 5H; Online Figure XVII). Interestingly, Arf1 was also activated in PH lungs, and its activity was reduced by SecinH3 (Figure 5F).

**Figure 6.**
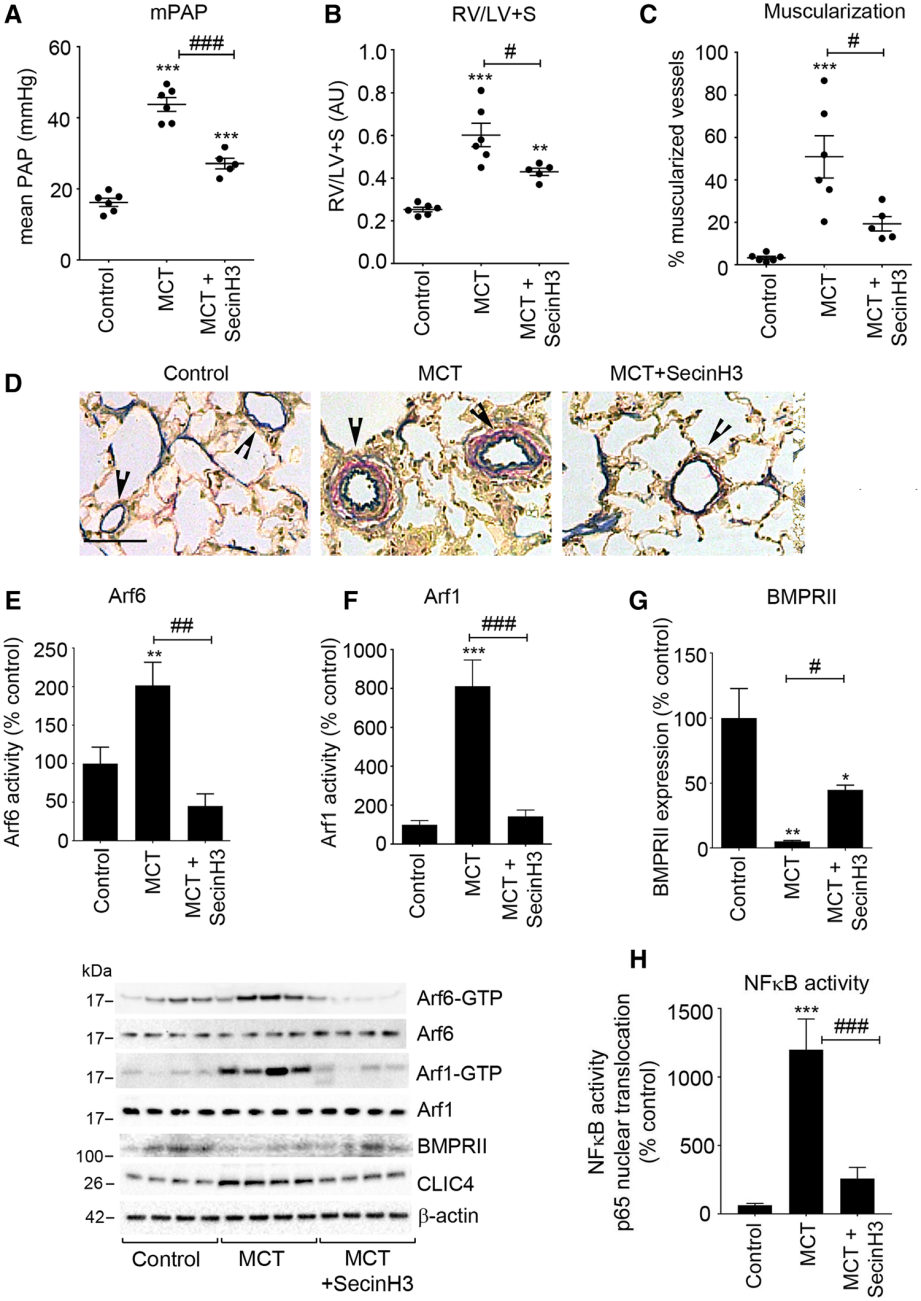
**Effects of Sec7 inhibitor H3 (SecinH3) on development of pulmonary hypertension, Arf activation, BMPRII, and CLIC4 expression in lungs of MCT rats.** (**A**) mPAP; (**B**) RV/LV+S; and (**C**) percentage of remodeled vessels in lungs of control and MCT rats treated with SecinH3, as indicated. **D**, Elastic van Gieson (EVG) staining showing fully muscularized peripheral arteries with double elastic laminae in MCT rat lung but single elastic laminae in control and SecinH3-treated rat lung (arrowheads). Bar=25 μm. **E**, Arf6 activity, (**F**) Arf1 activity, (**G**) BMPRII expression, and (**H**) NF-κB activity. Representative Western blots are shown below the graphs. *P<0.05, **P<0.01, ***P<0.001, comparisons with controls; #*P*<0.05, ##*P*<0.01, ###*P*<0.001 comparisons, as indicated. Data are presented as mean±SEM; n=6. One-way ANOVA with Tukey post hoc test. Arf6 indicates ADP ribosylation factor 6; BMPRII, bone morphogenetic protein receptor II; CLIC4, chloride intracellular channel 4; MCT, monocrotaline; and NF-κB, nuclear factor kappa B.

We next assessed the efficacy of SecinH3 treatment in the monocrotaline rat model of PAH, a nongenetic model of disease in which activation of NF-κB and reduction of BMPRII expression play a central role.^30,31^ The treatment regime started 8 days after a single injection of monocrotaline, when the pathological processes leading to vascular remodeling are already established.^32^ Increase in CLIC4 expression and Arf6 activation were noted from day 3, while significant rise in mean pulmonary artery pressure was noted later—14 days post-monocrotaline injection (Online Figure XVIII). SecinH3 significantly improved RVSP, RVH, and reduced arterial muscularization (Figure 6A through 6D; Online Figure XIX). These changes were accompanied by a marked reduction in Arf6, Arf1, and NF-kB activity and restoration of BMPRII expression without changes in CLIC4 expression in the lung (Figure 6E through 6H; Online Figure XX). SecinH3 did not cause any noticeable side effects or changes in the heart, liver, lung, kidney, spleen, or pancreas of treated animals (Online Figure XXI).

## Discussion

**Figure 7.**
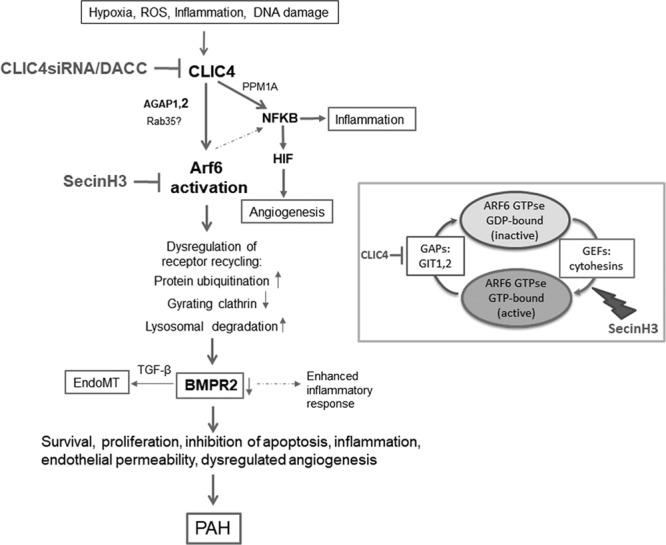
**Proposed CLIC4/Arf6 signaling pathway.** Arf6 indicates ADP ribosylation factor 6; BMPR, bone morphogenetic protein receptor; CLIC4, chloride intracellular channel 4; GAPs, GTPase-activating proteins; GEFs, guanine exchange factors; PAH, pulmonary arterial hypertension; PPM1A, protein phosphatase 1A; NF-κB, nuclear factor kappa B; and TGF-β, transforming growth factor.

This study shows for the first time that CLIC4 and Arf6 act together at the intersection of BMPRII and NF-κB signaling in PAH. We report a novel pathway through which BMPRII expression can be regulated, involving CLIC4/Arf6-dependent changes in receptor trafficking (Figure 7). We also demonstrate increased activity of CLIC4/Arf6 in ECFCs from idiopathic PAH patients and in lung tissues from Sugen/hypoxia mice and monocrotaline rats and show the effectiveness of CLIC4/Arf6 targeting in the treatment of the disease in 2 different preclinical models of PAH.

Dysfunctional endocytosis and recycling of cell-surface proteins is gradually being recognized as a hallmark of proliferative and inflammatory disorders, including atherosclerosis, PH, and malignant cancers.^23,33^ Once endocytosed into early endosomes, signal transduction receptors are either sorted into a recycling pathway that returns the molecules to the cell surface for another round of signaling or are sorted into a degradative pathway to be degraded in the lysosome.

Arf6, like other Arf proteins (Arf1-6), is regulated through the activation by GEFs and inhibition by GAPs. Physical interaction between CLIC4 and GIT proteins is likely to interfere with GIT-mediated GTP hydrolysis, resulting in the observed stabilization of an active, GTP-bound Arf6. *K*_*d*_ value for GIT1-CLIC4 interaction is in the low micromolar range, comparable with typical *K*_*d*_ values for ArfGAP-Arf interaction.^34^ As crystal structure of GIT proteins or the mechanism of GTP hydrolysis have not been fully characterized, the precise nature of CLIC4-GIT-Arf6 interaction will require further studies.

Our results indicate that CLIC4-induced inhibition of BMPRII signaling can be attributed to Arf6. Similarly, Arf6 but not Arf1 has been shown to terminate signaling of epidermal growth factor and luteinizing hormone receptors by shortening their lifetime at the cell membrane.^35,36^ Arf6-mediated internalization of membrane receptors and their subsequent targeting for lysosomal degradation can be prompted by proteolytic cleavage.^37^ Of relevance, Kaposi sarcoma-associated herpesvirus, which has been associated with the development of PAH, induces ubiquitination of BMPRII that leads to lysosomal degradation of the receptor.^4^ The precise contribution of CLIC4, which binds ubiquitin conjugation factor E4 A and alters expression of ubiquitin thioesterase ZRANB1, E3 ubiquitin-protein ligases RNF6 and TRAF7, to proteolytic degradation of membrane receptors will require further investigation.

Clathrin coat formation is governed by a complex web of reversible protein-protein interactions that engage under tight temporal and spatial regulation. Dysregulation of almost any component of the endocytic machinery often results in its complete collapse because of the unregulated sequestration of one or more interaction partners. Whether reduction in gyrating clathrin is a cause or a consequence of this dysregulation remains to be established. The association of CLIC4 with clathrin heavy chain or destabilization of Arf6 GTP-GDP exchange through interaction with GIT1 and GIT2 may reduce the levels of gyrating clathrin.^15^

Although CLIC proteins lack a recognizable signal sequence, they can induce chloride currents in artificial membrane systems under nonreducing conditions. This has led to the hypothesis that soluble CLICs can adopt an integral membrane conformation to form chloride channels under certain conditions. However, the channel hypothesis remains a matter of debate, and it seems likely that CLIC proteins have other cellular functions that are distinct from their proposed roles as chloride channels.^9^ While CLIC4 overexpression increased plasma membrane anion permeability in HPAECs, it is not clear whether this change was caused by CLIC4 or by subsequent activation of other transporters. The observed changes in anion conductance did not correspond to the changes in NF-κB activity or BMPRII expression, suggesting that anion conductance is not a major contributing factor. In addition, chloride channel blocker, indanyloxyacetic acid 94, did not affect CLIC4-induced changes in BMPRII expression. However, we cannot exclude the possibility that CLIC4 ion channel activity may affect immune responses in vivo. CLIC1^−/−^ macrophages show reduced phagocytosis, possibly associated with defective acidification of phagosomes.^38^ Consistent with the activation of lysosomal pathway, we observed increased numbers and acidification of lysosomes in CLIC4-overexpressing cells. Of interest, in endothelial cells, CLIC4 supports intravesicular acidification and vacuolar fusion through an unknown mechanism.^7^

CLIC4 increases serine phosphorylation of p65NF-κB^5^ required for its nuclear translocation, and we observed that serine phosphatase PPM1A had an inhibitory effect, reinforcing the view of potential involvement of this protein in CLIC4 signaling.^6^ Interestingly, we also observed that the pro-inflammatory effects of CLIC4 required the activity of Arf6. The exact mechanism is not known but may involve Arf6-mediated internalization of cytokine receptors.^39^ While silencing of BMPRII did not affect CLIC4-induced NF-κB activation in vitro, it is likely that some regulatory feedback mechanisms operate in vivo. BMPRII deletion reduces expression of superoxide dismutase in the vasculature,^40^ leading to increased ROS generation and DNA damage, known to stimulate CLIC4 expression.

The contribution of CLIC4/Arf6 pathway to vascular dysfunction is likely to extend beyond the regulation of BMPRII and NF-κB signaling. Both CLIC4 and Arf6 play regulatory roles in pathways enhancing cell proliferation, migration, increase endothelial permeability, angiogenesis, and are predictors of poor prognosis in cancer.^5,41–43^ Accordingly, Arf6 siRNA and SecinH3 inhibited CLIC4-induced HIF activation and angiogenic responses in cultured HPAECs. CLIC4 and Arf6 are activated by angiotensin II^44,45^ and mediate the effects of TGF-β.^6,46^ We noted a marked upregulation of CLIC4 and Arf6 signaling in ECFCs from idiopathic PAH patients (5- and 10-fold increase, respectively), suggesting potential importance of this pathway in the human condition. ECFCs represent an accessible surrogate cell type to study endothelial dysfunction in PAH and display several characteristics of the disease, including reduced BMPRII expression, disorganized angiogenesis, increased permeability, increased HIF and NF-κB activation, which also represent responses augmented by CLIC4/Arf6 signaling.

Importantly, we show that CLIC4 expression in the lung vasculature can be targeted using the DACC siRNA delivery system. DACC/CLIC4 siRNA treatment reduced Arf6 but not Arf1 activity, restored BMPRII expression, and attenuated the development of PH in Sugen/hypoxia mice. These experiments confirm a critical role of CLIC4/Arf6 pathway in disease pathogenesis and demonstrate therapeutic potential of gene targeting by RNA interference. Further, we show that pharmacological targeting of CLIC4/Arf6 signaling with SecinH3^25^ prevents CLIC4-induced effects in vitro (inset in Figure 7) and attenuates development of PH in Sugen/hypoxia mice and monocrotaline rats. SecinH3 was developed as a specific inhibitor of small sec7 containing GEFs such as Arf-GEFs, which prevents Arf6 activation by inhibiting GDP for GTP exchange with dissociation constants (*K*_*d*_ values) between 200 and 250 nmol/L.^25^ Preclinical studies show that SecinH3 and other inhibitors of cytohesins are effective in treatment of inflammatory and hyperproliferative diseases. While we did not observe any detrimental effects of SecinH3 treatment in our study, cytohesin inhibitors were shown to increase insulin resistance in mice.^25^ Antiproliferative effects of SecinH3 correlate with a profound inhibition of Akt activation and survivin expression in lung cancer.^47^ SecinH3 has also been shown to reduce lung injury in septic rats and increase vascular stability in mouse models of arthritis.^48,49^ In addition to the inhibition of Arf6, which localizes to the plasma membrane, SecinH3 may also inhibit the activity of Golgi-localized Arfs (Arf 1,3,4,5). Activation of Arf1 in PH lungs is likely to reflect the importance of Golgi-dependent protein trafficking in vascular remodeling. The role of Arf-regulated Golgi dynamics and the effect of cytohesin inhibitors on trafficking of other membrane receptors involved in PH pathogenesis will require further studies.

In summary, we identify a novel pathway involving activation of the endocytotic trafficking regulator, Arf6. Inhibition of CLIC4/Arf6 pathway represents a novel strategy in treatment of PAH.

## Acknowledgments

We thank the staff of the NIHR/Wellcome Trust-Imperial Clinical Research Facility, Hammersmith Hospital (London, UK) for their help and Professor James Keen, Thomas Jefferson University, PA, for gift of YFP-GGA1 and Professor Xin-Hua Feng, Baylor College of Medicine, Houston, TX, for pRK5F-PPM1A. We also thank Professor Stuart H. Yuspa (Laboratory of Cancer Biology and Genetics, Centre for Cancer Research, Bethesda) for adenoviral CLIC4 construct and Professor John C. Edwards (Division of Nephrology, Department of Internal Medicine, St. Louis University, St. Louis, MO) for helpful discussions regarding chloride channel activity of CLIC4.

## Sources of Funding

This research was supported by project grants PG/15/69/31719 and PG/16/4/31849 from the British Heart Foundation.

## Disclosures

None.

## Supplementary Material

## Novelty and Significance

### What Is Known?

- Reduced expression of BMPRII (bone morphogenetic protein receptor II) is a feature of occlusive vasculopathy in pulmonary arterial hypertension (PAH).
- BMPRII degradation is increased in PAH.
- CLIC4 (chloride intracellular channel 4) contributes to vascular remodeling in PAH, but its effect on BMPRII function is not known.
