# Supplementary material for: CLIC4/Arf6 Pathway: A New Lead in BMPRII Inhibition in Pulmonary Hypertension
Source: Circ Res. 2018 Oct 15;124(1):52–65. doi: 10.1161/CIRCRESAHA.118.313705 (PMC6325770; doi:10.1161/CIRCRESAHA.118.313705)
Supplement: Supplementary file 1 [file res-124-52-s001.pdf]

# SUPPLEMENTAL MATERIAL

## SUPPLEMENTAL MATERIALS AND METHODS

### Proteomic analysis

**Purification of HA-tagged protein.** HA-tagged protein purification kit (3320, MBL Company Ltd) was used for purification of proteins bound to HA-tagged CLIC4. 24hr post-infection with AdCLIC4 or AdControl, HPAECs were lysed and the lysates (500 µL each) were transferred into two separate micro centrifuge tubes. Anti-HA tag beads suspension (40 µL containing 10 µL beads) was dispensed into each tube and was incubated with gentle end-over mixing for 1hr at 4°C. The tubes were then centrifuged (3000g at 4°C) for 20s and the resultant supernatant containing non-binding proteins was removed. The beads were washed three times with 500 µL PBS where the mixture was centrifuged (3000g at 4°C) for 20s and the supernatant removed each time. Finally, elution peptide solution (20 µL) was added and incubated with the beads for 5min at 4°C. The tubes were centrifuged again and the supernatant containing interacting proteins was reserved for further analysis.

**Preparation of samples for LC-MS/MS analysis.** Cell homogenates or fraction containing purified proteins were dissolved in 300 µL of 9 mol/L urea and reduced by addition of 50 mmol/L dithiothreitol prior to heating at 100°C for 2min and then carbamidomethylated by addition of 200 mmol/L iodoacetamide in the dark for 30min. SDS-PAGE was performed on 10 µg of each sample using 10% NuPAGE Novex bis-tris gels and reagents (Invitrogen). Gels were stained with InstantBlue® and each sample-containing lane were cut into a series of regions based on the position of molecular weight (MW) markers (SeaBlue Marker®, Invitrogen) and the distribution of proteins observed in the gel. Gel pieces were then washed with 1 mL water and dehydrated in 1 mL acetonitrile for 30min. Proteins contained within each gel slice were digested in 300 µL of 50 mmol/L ammonium bicarbonate containing 1 ng/mL trypsin (sequencing grade, Promega) at 37°C for 18hr. On the following day, tryptic peptides were extracted by addition of 300 µL 0.1% formic acid in 2% acetonitrile and mixed for 30min, 420 µL was removed and dried.

**LC-MS/MS analysis method.** The dried samples were reconstituted in 30 µL of 0.1% TFA and 8 µL of the solution was injected onto a C18 trap-column (ProteCol, 0.3 x 10 mm, 300Å; SGE Analytical Science) followed by reverse phase separation on a C18 column (PicoFrit, 75µm ID × 10 cm ProteoPrep column, New Objective) using Agilent 1200 LC series (Agilent Technologies). The mobile phases were 0.1% formic acid in water (buffer A) and 0.1% formic acid in acetonitrile (buffer B). A linear gradient of 0-54% buffer B was introduced at a flow rate of 300 nL/min for 48min. The peptides eluting from the reversed phase column were analysed on-line using a Thermo LTQ linear ion trap MS equipped with a dynamic nano-spray interface. The MS method consisted of a cycle combining one full MS scan (m/z 400-1600) with three data dependent MS/MS events (35% collision energy at each event) for 70min. Dynamic activation time was set at 30s with scans at every 2µs.

**MS data analysis.** MS data was assessed on the basis of ion intensity of peptide ions with coincident LC retention time and m/z values. These data were displayed as an intensity map by Progenesis LC-MS software (Nonlinear Dynamics). The software aligns data based on the LC retention time of each analysis to create a single aggregate run containing all of the MS data with those representing peptide ions indicated. Feature outline maps were then generated and these outlines were used to facilitate detection and quantification of peptide ions from individual analyses. The peptide quantitation algorithm in the software reports peptide abundance as the sum of the peak areas (pixel intensities) within its isotope boundaries. Each abundance value is then transformed to a 'normalised' abundance by applying a global scaling factor calculated from distribution factors of all peptide ratios in the analysis. This corrects for experimental/technical variation and ensures that up-regulation and down-

regulation has the same weight. The distribution is calculated using log (ratio) values and as log (1) = 0.0 and can be modelled as follows: $y'_i = \alpha_k y_i$ . Protein abundances for individual sample are then calculated as the sum of all component peptide abundances.

The identity of peptides was determined by exporting the MS/MS output files from the matched analysis into the Turbo SEQUEST search engine (BioWorks Browser 3.3, Thermo Electron Corporation) and interrogating it against the NCBI human RefSeq database release 51. The search parameters were as follows: peptide tolerance was set to 2 atomic mass unit, MS/MS tolerance was set at 1 atomic mass unit, charge states allowed is from +1 to +3 and up to two missed cleavages were allowed. Modifications were set to allow for the detection S-carbamidomethylated cysteine (+57 Da). Each MS2 spectra was matched to only a single peptide with the highest scoring SEQUEST peptide hit. Cross-correlating (XCorr) MS data, with values >1.5, >2.0 and >2.5 for singly, doubly and triply charged ions respectively, were used to select peptides of high quality and reduced the possibility of including redundant peptides in subsequent data analysis.

All keratins were removed from the protein identification list. The current status of each protein, its name and molecular weight was updated by batch processing it on the NCBI protein database website. Where protein names contained the word ‘predicted’ or ‘like protein’, the accuracy of the assigned names were examined and corrected, where necessary. The issue of redundancy in identified proteins was addressed by including all possible assignments in the results table to make the data as transparent as possible. In those cases where an identified protein shares its sequence with other entries in the database then all entries are listed. The uniqueness (or otherwise) of the assignment of peptides to the sequence of one or more proteins was used to determine whether assignment could be made to a specific protein or to a group of related proteins (isoforms). Quantification was based on at least 2 peptides that were all uniquely assigned to one protein or to a group of related forms.

Protein abundance values were calculated on the basis of the ion intensities of the component peptides. For detection of differentially expressed proteins, only proteins identified on the basis of at least two unique peptides were considered. For interacting proteomics analysis, only proteins that were unique to the HA-tag purification fraction are listed. For overexpression analysis, differentially expressed proteins were identified on the basis 1.5-fold difference in abundance in AdCLIC4 versus AdControl and P-value <0.05. Ingenuity Pathway Analysis was used for mapping the identified proteins to known biological pathways.

The mass spectrometry proteomics data have been deposited to the ProteomeXchange Consortium via the PRIDE[1] partner repository with the dataset identifier **PXD008709** for “CLIC4 interacting proteins” and **PXD008714** for “Proteomics of CLIC4 overexpressing human pulmonary artery endothelial cells”.

**Cell transfection.** Electroporation of plasmid DNA with BMPRII-GFP (RG208673, Origene), pRK5F-PPM1A (protein phosphatase 1A plasmid DNA, kind gift of Professor Xin-Hua Feng, Baylor College of Medicine, Houston, Texas, USA), pmaxGFP™ (Lonza), YFP-GGA1or mCherry clathrin[2211] (kind gift of Professor James Keen, Thomas Jefferson University, Philadelphia, USA), Silencer Select human siRNAs: negative control siRNA#1 (4390843, Ambion), Arf1 siRNA (4390824, ID: s1552, Ambion), Arf6 siRNA (4390824, ID: s1565, Ambion) were conducted using the Amaxa™ Basic Nucleofector Kit (VPI-1001, Lonza). Briefly, 80% confluent HPAECs were trypsinised with 0.05% trypsin-EDTA and centrifuged at 400g in the presence of culture medium containing 10% FCS. The pellet containing 5 X $10^5$ cells was then re-suspended in a certified cuvette in 100 µL Nucleofector™ Solution containing 2 µg plasmid DNA or 2 pmol (100 nmol/L) of siRNA. The cuvette was then transferred to the Nucleofector™ 2b Device (AAB-1001, Lonza) and electroporation was then conducted using the M-003 program. Transfected HPAECs were then cultured on Thermanox® Plastic Coverslips (13 mm; 174950, Thermo Scientific) in 24 well culture plates, or 96 well cell culture plates (10,000 cells/well; 83.3924, Sarstedt). In experiments using DNA plasmids, 3hr post transfection, the cells were left untreated or were infected with AdCLIC4/AdTet-off, with or without AdNFkB, as appropriate. The cells where allowed to overexpress the recombinant proteins for 24hr before immunostaining or the luciferase

reporter assay. In experiments involving siRNA, the cells were infected with Adcontrol or AdCLIC4/AdTet-off 24hr post-transfection and were used for experiments on the following day (48hr post-transfection).

In some experiments, transfection with silencing RNAs was carried out using lipofectamine. Briefly, endothelial cells were seeded in 6-well plates (200,000 cells per well). The following day, siRNA duplexes (50 pmol per well) specific for Arf6 or Arf1 or a scrambled sequence serving as control siRNA were transfected in OPTI-MEM medium (Invitrogen) using Lipofectamine RNAiMAX (13778075, ThermoFisher Scientific), according to the manufacturers optimised general protocol. The efficiency of the knockdown was determined 48hr after transfection by measuring protein levels in cell extracts, using western blotting.

**Immunostaining and western blot analysis.** CLIC4, p65 NFκB, BMPRII, Smad 1,5 and p-Smad1,5, Smad 3 and p-Smad 3, Arf1, Arf6, LAMP-1, β-actin and F-actin, were studied by western blotting and immunofluorescence in cells, cell lysates or tissue sections, as appropriate. For immuno- and affinity-fluorescence analysis, HPAECs grown on Thermanox coverslips were fixed with 4% formaldehyde in PBS, permeabilised with 0.1% Triton X-100 in PBS, incubated with 2% bovine serum albumin (Sigma) in PBS for 1hr and then incubated with mouse monoclonal anti-CLIC4 antibody (1:100; sc-135739, Santa Cruz Biotechnology), mouse monoclonal anti-HA antibody (1:100; sc-805, Santa Cruz Biotechnology), rabbit anti-BMPRII antibody (1:100; sc-20737, Santa Cruz Biotechnology), or rabbit anti-LAMP1 antibody (1:100; ab24170, Abcam). Fluorescently-labelled secondary antibodies used for immunostaining included TRITC-Goat Anti-Rabbit Ig (1:200; 111-025-144, Jackson Immuno Research Laboratories), Cy5™ Goat anti-Mouse IgG (1:200; 81-6516, Zymed® Laboratories), FITC-Goat Anti-Mouse IgG (1:200; 115-095-003, Jackson ImmunoResearch Inc.) and F-actin was visualized with 1 µg/ml TRITC-phalloidin (P1951, Sigma). Following immunostaining, cells were mounted in Vectashield with DAPI and examined under the fluorescent confocal microscope (Leica, TCS SP5, Leica Biosystems, Bretton, Peterborough, UK).

Primary antibodies used in western blotting included: mouse monoclonal anti-CLIC4 (1:1000; sc-135739, Santa Cruz Biotechnology), mouse monoclonal anti-BMPRII (1:500; 612292, BD Bioscience), rabbit anti-Phospho-Smad1/5 (Ser463/465) (1:500; 9516, Cell Signalling Technology), rabbit anti-smad1 (1:1000; 9743, Cell Signalling Technology) rabbit anti-smad5 antibody (1:1000; 9517S, Cell Signalling Technology), mouse monoclonal anti-β-actin (1:10000; A1978, Sigma-Aldrich) and secondary antibodies HRP-linked goat Anti-Rabbit IgG (1:3000; A6154, Sigma-Aldrich) and HRP-linked sheep anti-mouse IgG (1:1000; NAG31V, GE Healthcare Life Sciences).

The relative intensity of the immunoreactive bands was determined by densitometry using Image J software (Rasband, W.S., ImageJ, U. S. National Institutes of Health, http://imagej.nih.gov/ij/, 1997-2011).

For immunostaining of paraffin embedded lung sections, the slides were dewaxed and rehydrated prior to a heat-induced antigen retrieval in 10 mmol/L sodium citrate (pH 6.0) and 0.05% Tween 20, at 80°C for 20min. Sections were incubated with a mouse monoclonal anti-CLIC4 antibody (20 μg/mL; sc-135739, Santa Cruz Biotechnology) or a polyclonal rabbit antibody raised against human von Willebrand Factor (10 μg/mL; A082, Dako;). Controls included the use of affinity purified non-immune rabbit IgG instead of the primary antibodies. Sections were then stained using the avidin-biotin-peroxidase complex (ABC Elite, Vector Laboratories) method with 3,3'-diaminobenzidine as a substrate.

**Tissue homogenate preparation and western blotting analysis.** For western blot analyses, tissue samples were homogenised in RIPA buffer (Sigma) for 1min using PT-K Polytron® Stand Homogenizer (Kinematica AG). Samples were kept on ice throughout the procedure and frozen at -80°C in aliquots of 100 µL until analysed. For Arf6 activity analyses, tissue samples were homogenised in a lysis buffer

from the Arf6 activity assay kit (BK033-S, Cytoskeleton, Inc.). Protein content was estimated using the bicinchoninic acid method (Thermo Fischer Scientific) with bovine albumin as standard.

Protein expression of CLIC4, BMPRII, Arf1, Arf6 and β-actin in the lung tissues was examined by western blotting. Proteins were separated by 4-12% NuPAGE® Bis-Tris gels (Invitrogen) using MES buffer at 200V constant for 35min (5 µg equivalent of protein/lane). Membranes were probed with a mouse monoclonal antibody raised against a synthetic peptide sequence (238-250 amino acids) of human CLIC4 (0.2 µg/mL; s c-135739, Santa Cruz Biotechnology). Controls included the use of mouse monoclonal antibodies against β-Actin (1:10,000; A2228, Sigma). The relative intensity of the immunoreactive bands was determined by densitometry using Image J software (Rasband, W.S., ImageJ, U. S. National Institutes of Health, Bethesda, Maryland, USA, http://imagej.nih.gov/ij/, 1997-2011).

**Microscale Thermophoresis (MST).** MST was performed with human kidney embryonic cells (HEK) 293 cell lysate containing Flag-ECFP-GIT1 (15223, Adgene, depositing lab Dr Rick Horowitz) and a serial dilution of purified CLIC4 to assess the interaction between the two proteins[3,4]. HEK293 cells (85120602, Sigma) grown in T75 flasks (156499, ThermoFisher Scientific) in Dulbecco Modified Eagle Medium (DMEM; 10313-02, Gibco) supplemented with 10% foetal bovine serum (Sigma) with antibiotics, were transfected with pCDNA3 Flag-ECFP-GIT1 using Lipofectamine 2000 (11668027, ThermoFisher Scientific) with 5 µg ECFP-GIT1/15 µL Lipofectamine 2000/1x10$^6$ cells, according to the manufacturer's protocol (https://tools.thermofisher.com/content/sfs/manuals/Lipofectamine_2000_Reag_protocol.pdf). 24hr post-transfection, the cells were lysed in PBS containing 0.1% (v/v) Tween-20 (P9416, Sigma) and cOmplete™ protease inhibitor cocktail (11697498001, Sigma). The lysates were cleared by centrifugation at 15,000g at 4°C for 5min and stored at -80°C. Before MST assay, the lysates were thawed and centrifuged at 20,000g at 4°C for 20min to remove precipitate. The lysates were then diluted in 20 mmol/L Tris-HCl, pH 7.4, 150 mM NaCl, 10% (v/v) glycerol; supplemented with cOmplete™ protease inhibitor cocktail (11697498001, Sigma) to yield 200 counts of fluorescent signal at 20% LED power (equivalent to 20 nmol/L concentration) as judged by the Monolith NT.115 instrument according to the manufacturer's protocol (NanoTemper Technologies, Germany). The assay was carried out with constant concentration of ECFP-labelled GIT1 at 20 nmol/L with varying concentrations of CLIC4. Sixteen tubes of a two-fold serial dilution of CLIC4 covering a concentration range of 0.24-8000 nmol/L was prepared. An equal volume of ECFP-GIT1 lysate (diluted to 200nmol/L) was added to each CLIC4 concentration in the dilutions series, and incubated at room temperature for 15min before loading into premium MST capillaries. Single MST experiments were performed using a Monolith NT.115 instrument with an LED power of 80% and MST power of 80% with a wait time of 5s, laser on time of 20s and a back-diffusion time of 5s. NanoTemper Analysis 1.2.101 software was used to analyse raw data to obtain normalized fluorescence, which was subsequently plotted in GraphPad Prism. The CLIC4-GIT1 binding experiment was repeated at least three times, and the BSA control was repeated twice. The mean apparent binding affinity ($K_d$) values were calculated with Standard Error of Mean (SEM) in GraphPad Prism using a specific binding equation.

**Arf6 and Arf1activity assays.** Arf6 and Arf1 activity assays were carried out according to the manufacturer's protocol (BK033-S and BK032-S, respectively; Cytoskeleton, Inc.). Briefly, cells or tissues were lysed in 800 µL of the lysis buffer provided and then centrifuged for 3min at 10,000g. The supernatants were used as follows: 700 µL were transferred to a fresh tube containing 20 µL of GGA3-PBD beads, 20 µL were used for determination of protein concentration and 30 µL were mixed with 10 µL of Laemli buffer with reducing agent for the determination of total Arf6 or Arf1 expression. The supernatants were incubated with the GGA3-PBD beads with rotation for 1hr at 4°C. The beads were

then centrifuged at 500g for 2min at 4°C and the pellets were washed with 600 µL of wash buffer three times – with similar pelleting method in each washing step. After the final wash, 20 µL of Laemli buffer with reducing agent were added to the pellet and the activity of Arf6 or Arf1 were determined by western blotting using Arf6 or Arf1 antibody (sc-7971 and sc-53168, respectively, Santa Cruz Biotechnology).

**Quantification of Gyrating (G-) clathrin.** HPAECs transfected with plasmid encoding YFP-GGA1 were infected with Adcontrol or AdCLIC4 and incubated with, or without SecinH3 (10 mg/L), as appropriate. Twenty-four hours later, images of live cells were recorded under the confocal laser scanning fluorescence microscope (Zeiss LSM-780 at the facility for imaging by light microscopy; FILM) using a 40x 1.3na objective for 58s at a frame rate of 0.39s per frame to collect frame image stacks. For quantification of YFP-GGA1 in ImageJ, the first 50 frames for each channel were selected, the background subtracted using the rolling ball command and photo-bleaching over time corrected using bleach correction command. In each time series, a maximum projection image (comprising both highly mobile G-GGA1 and stable structures) and sum projection image (emphasizing stable structures) of each stack, were prepared. Each stack was thresholded and binarized. Regions 80 pixels on edge were randomly selected near the periphery of the cell. The ratio of the maximum projection pixel area to the sum pixel area in a single image yielded an estimate of 'G-GGA1 area'. Approximately 30 regions for each condition in three independent experiments were analysed, and mean G-GGA1 area in control cells was set to 100%. Data were analysed by ANOVA. In addition, to visualise trajectories of YFP-GGA1 vesicles, stack images were analysed with TrackMate in Image J. Stacks were background corrected using a rolling ball filter (r=5) in ImageJ. Using MetaMorph (Molecular Devices Inc.), a maximum projection (comprising) and sum projection emphasizing unwavering (stable) structures of each stack, were prepared.

**NFκB luciferase reporter assay and nuclear translocation of p65NFκB.** HPAECs were infected with AdNFκB-Luc (Vector Biolabs) at a MOI of 1:500. NFκB-driven luciferase expression was measured 20hr post-transfection using a Luciferase Assay System (Promega) and GloMax®-Multi Microplate Multimode Reader. Nuclear translocation of p65 NFκB was studied with Image J by measuring colocalization of nuclear stain DAPI with NFκB stained with rabbit p-NFκB p65 (Ser 276) (1:200; sc-101749, Santa Cruz Biotechnology) and secondary antibody, TRITC-labelled goat anti-rabbit antibody (1:200; 111-025-144, Jackson ImmunoResearch Laboratories,) in confocal images of cells or lung sections. The white pixel area, marking nuclear NFκB, was used to quantitate p65 NFκB translocation in cells and tissues.

**Proximity Ligation Assay (PLA).** Intracellular distribution and spatial proximity of CLIC4 and BMPRII in HPAECS was studied with Duolink in situ PLA according to manufacturer's instructions (Olink Biosciences; Sigma-Aldrich). HPAECs grown on Thermanox coverslips inserted into 24-well TC plates (83.3922, Sarstedt) were infected with either Ad-tet-off or AdCLIC4. In some experiments, to allow visualization of lysosome marker LAMP1, 6 µL of CellLight® Lysosomes-GFP BacMam 2.0 reagent containing baculoviral LAMP1 overexpression vector (C10596, Thermo Fisher Scientific ) was added to ~ 3 x$10^5$ cells/well. 24hr post-transfection, HPAECS were fixed with 4% paraformaldehyde and permeabilised with 0.1% Triton X-100. HPAECS were incubated with Duolink blocking buffer for 30min at 37°C then incubated overnight with rabbit anti-CLIC4 (1:100; HPA008019, Sigma) and mouse anti-BMPR2 (1:50; BD Transduction Laboratories). HPAECS were then incubated with anti-rabbit PLUS and anti-mouse MINUS PLA probes (1:5) for 1hr at 37°C and then ligated using the provided ligase-ligation solution for 30min at 37°C. Samples were then amplified with Duolink In Situ Detection Reagent Orange for 100min at 37°C. Samples were mounted with Duolink mounting media and images were taken under a fluorescent confocal microscope (Leica, TCS SP5, Leica Biosystems, Bretton, Peterborough, UK). The number of fluorescent puncta, indicative of a close apposition of the two proteins, was scored by measuring the intensity of fluorescence in each cell with Image J.

**Clathrin-mediated endocytosis.** Clathrin-mediated endocytosis was evaluated by measuring fluorescence intensity of transferrin from human serum, Alexa Fluor™ 488 Conjugate (T13342, Thermofisher Scientific), taken up by cells. Briefly, ~70% confluent HPAECs grown on coverslips were incubated in serum-free EGM-2 for 45min. Then, 30 mg/L fluorescent transferrin and 20 µg/ml albumin (Sigma) were added to the cells and incubated for further 30min at 37ºC. The cells were then washed in PBS, fixed in 4% formaldehyde for 15min and mounted in Vectashield. Confocal images were analysed in Image J. To evaluate clathrin-independent endocytosis, cells were incubated with 10 mg/L mouse monoclonal antibodies directed towards major histocompatibility complex-I (MHC-I) (clone w6/32, Biolegend) or control, mouse monoclonal anti-cytokeratin 14 antibody (ab7800) (directed against intracellular target) at 37°C for 30min to allow endocytosis of the antibody. After internalization, surface antibody was removed by low pH acid wash (0.5% acetic acid in 0.5 M NaCl for 30s) and cells were fixed in 4% formaldehyde in PBS, incubated with unlabelled goat-anti mouse IgG in the absence of detergent for 10min. Then the cells were washed 3x in PBS, permeabilised in 0.1% Triton X-100 solution in PBS for 5min, blocked in 5% bovine serum albumin (Sigma) in PBS for 30min, incubated with 10 mg/L of secondary, FITC-labelled goat anti-mouse antibody (115-095, Jackson Laboratories) for 1hr, washed 3 times in PBS, mounted in Vectashield with DAPI and examined under the fluorescent confocal microscope (Leica, TCS SP5, Leica Biosystems, Bretton, Peterborough, UK).

In some experiments, control (Adcontrol) and AdCLIC4-overexpressing (AdCLIC4) cells were treated with 20 mg/L protein synthesis inhibitor cycloheximide (CAS 66-81-9, Santa Cruz Biotechnology), with, or without the endocytosis inhibitor Pitsop2 (20 µmol/L; ab120687, Abcam) or Pitstop2 negative control (20 µmol/L; ab120688, Abcam) for 2hr. Pitstop2 negative control is a compound chemically related to Pitstop2 which does not block receptor-mediated endocytosis. The cells were then lysed and changes in BMPRII expression were analysed by western blotting.

**HIF activation and tube formation assay.** HIF-1α stabilization was studied in human osteosarcoma cells (U2OS) stably expressing a luciferase reporter construct under the control of a hypoxia response element (U2OS-HRE-luc) in control and CLIC4-overexpressing cells transfected with control siRNA, Arf1siRNA, Arf6 siRNA or treated with SecinH3 and cultured in normoxic or in hypoxic conditions (2% $O_2$, 5% CO2, 92% N2) for 24hr. Briefly, 24hr post-transfection with silencing RNAs, U2OS cells were left either untreated (control), were infected with the tet-off adenovirus (AdControl) or with CLIC4 and tet-off adenoviruses (AdCLIC4) to allow expression of CLIC4. The cells were then incubated under normoxic or hypoxic conditions for 24hr and a luciferase assay (E1500, Promega) was used to measure HIF activation.

Angiogenic responses were studied in the matrigel tube formation assay in control and CLIC4-overexpressing HPAECs transfected with negative control (non-targeting) siRNA, Arf1 siRNA or Arf6 siRNA or treated with SecinH3 and cultured in normoxic or in hypoxic conditions for 24hr. Briefly, 24hr post-transfection with silencing RNAs, the cells were infected with AdControl or AdCLIC4 adenoviruses and 4hr later the cells were trypsinized, re-suspended in growth factor-deprived media containing 1% FBS and seeded at a density of 7000 cells/well in 96 well plates coated with 50 µl Matrigel (354230, BD Biosciences). Plates were then incubated for 24hr, under normoxic or hypoxic conditions. Live cells were then fluorescently labelled with Cell Tracker Green before examination under a fluorescent microscope. Fluorescent and phase-contrast images were captured from each well under (10x) objective and total tube length was determined using Image J software. In HIF activity and tube formation assay 4 wells/treatment in 4 experiments were analysed.

**Plasma membrane anion permeability.** Cell plasma membrane anion permeability was assessed using the Chloride Channel Assay Kit from Abcam (ab176767) which measures passive cellular iodide uptake, according to the manufacturer's protocol. Briefly, HPAECs were plated in 96-well dishes at the density of $4x10^4$ cells/well, incubated with loading buffer containing potassium iodide for 5min, washed with PBS, lysed and the lysates were incubated with a colorimetric iodide indicator. The 630 nm absorbance, proportional to the intracellular concentration of potassium iodide, was measured with GloMax®-Multi Microplate Multimode Reader.

**Animal experiments.** All experiments were conducted in accordance to the UK Home Office Animals (Scientific Procedures) Act 1986 (London, UK).

All animals were randomly allocated to groups, and all personnel involved in data collection and analysis (hemodynamics and histopathologic measurements) were blinded to the treatment status of each animal. Only weight-and age-matched males were included for experimentation as, in contrast to the human clinical studies, most animal studies have shown that female sex and estrogen supplementation have a protective effect against PAH.

In Sugen/hypoxia mouse model of PH, adult male C57/Bl6 mice (20g, 8/group) were injected subcutaneously with Sugen (SU5416; 20 mg/kg, Tocris Bioscience), suspended in 0.5% [w/v] carboxymethylcellulose sodium, 0.9% [w/v] sodium chloride, 0.4% [v/v] polysorbate 80, 0.9% [v/v] benzyl alcohol in deionized water once/week. Control mice received only vehicle. The animals were exposed to chronic normobaric hypoxia (10% $O_2$) in a ventilated chamber for 21 days. DACC/CLIC4siRNA or DACC control non-targeting siRNA were delivered via iv injection (2.8 mg/kg body weight) twice a week. First injection was given 1 day before Sugen/hypoxia administration. The following day the mice were injected with Sugen and exposed to hypoxia for 3 weeks (n=8/group). Single injection of fluorescently labelled DACC/siRNA-Cy3 (2.8 mg/kg body weight) in healthy mice was used to follow distribution of siRNA in lungs, heart, liver and kidney 4hr and 24hr post-injection. Localization of the fluorescent mimic to the endothelium was confirmed by co-immunostaining for von Willebrand Factor (vWF).

To prepare siRNA lipoplexes, cationic liposomes comprised of cationic lipid AtuFECT01 (β-L-arginyl-2,3-Ldiaminopropionic acid-N-palmityl-N-oleyl-amide trihydrochloride; Silence Therapeutics GmbH, Berlin, Germany), cholesterol (Sigma Aldrich, Taufkirchen, Germany), and mPEG2000-DSPE (1,2-distearoyl- sn-glycero-3-phosphoethanol amine-N (methoxy (polyethylene glycol)- 2000); Avanti Polar Lipids, Alabaster, AL) at a molar ratio of 70:29:1 were prepared by lipid film rehydration with 270 mmol/L sterile sucrose solution. The resulting liposomal stock solutions had total lipid concentrations of 5 mg/ml or up to 9 mg/ml (i.e., for infusion studies), respectively. The formation of siRNA lipoplexes occurred by mixing equal volumes of liposomal dispersion and siRNA solution in 270 mmol/L sucrose. For this purpose, the concentration of both were adjusted in a way, that the final lipoplex formulation was characterized by a final lipid/siRNA ratio (m/m) of 6.8, which corresponded to a charge ratio between charged lipid nitrogen atoms to nucleic acid backbone phosphates (N/P ratio) of approximately 8.4. Particle sizes (Z-average size, intensity distribution) and zeta potentials of liposomes and lipoplexes were determined by Dynamic Light Scattering using a Zetasizer Nano-ZS (Malvern Instruments, Worcestershire, UK). Here, corresponding dispersant properties were adjusted to 270 mmol/L sucrose. Negative stain transmission electron microscopy was done by Vironova AB (Stockholm, Sweden).

In the second prevention strategy, Sugen/hypoxia mice (n=8/group) were injected every other day intraperitoneally either with vehicle (20% DMSO and 10% Tween-20) or SecinH3 (100 μL of 5 mmol/L; 2849, Tocris Bioscience) dissolved in vehicle. The normoxic control group received vehicle only. Following 21 day hypoxic exposure, the animals were removed from the hypoxic chamber individually and anaesthetised by IP injection of Ketamine/Dormitor (75 mg/kg + 1 mg/kg). Mice were assessed for development of pulmonary hypertension by measuring right ventricular systolic pressure (RVSP), right ventricle to left ventricle/septum ratio (RV/LV+S) and pulmonary vascular remodelling.

In the third series of experiments, male Sprague-Dawley rats (190–200 g, n=6/group) were injected subcutaneously with a single dose of monocrotaline (60 mg/kg body weight). Monocrotaline was dissolved in 1 mol/L HCl, diluted with saline and neutralized to pH 7.4 with 10 mol/L NaOH to prepare a 40 mg/mL solution. One week after MCT injection, six rats were injected intraperitoneally every other day with vehicle (20% DMSO and 10% Tween-20), while the other group (n=6) was injected with SecinH3 (2.5 mg/kg body weight) dissolved in vehicle, for a total of 14 days. Control rats (no MCT) were injected with vehicle intraperitoneally every other day. After 21 days of the study, rats were anaesthetised with fentanyl/fluanisone (Hypnorm, VetaPharma) using intramuscular injection at 1 ml/kg

body weight; followed by intraperitoneal injection with 0.8 mL/kg body weight of midazolam (Hypnovel, Roche). Haemodynamic parameters including mean pulmonary artery pressure (mPAP) and RVSP were measured using a pre-curved catheter inserted through the right jugular vein, passed by the right ventricle into the pulmonary artery. Additionally, a group of control rats (n=4/group) were either treated with vehicle or SecinH3 (2.5 mg/kg body weight), for a total of 14 days to evaluate the effect of SecinH3 on major rat organs. To evaluate changes in CLIC4 and Arf6 expression in disease development, male Sprague-Dawley rats (190–200 g, n=4/group) were injected subcutaneously with a single dose of MCT (60 mg/kg body weight), as above. The haemodynamic changes and levels of relevant proteins were measured at 3, 7 and 14 days post-MCT injection.

In all studies, the degree of right ventricular hypertrophy was determined by calculating the weight/weight ratio of the right heart chamber (RV) to the left heart chamber with septum (LV+S). Right lung lobe was harvested and snap frozen in liquid nitrogen while the left lobe was inflation-fixed (10% formaldehyde in PBS), embedded in paraffin, and sectioned for histology. Heart, liver, kidney, spleen and pancreas were collected, fixed, paraffin embedded and stained with H&E to assess the effect of SecinH3 treatment. Transverse formalin-fixed lung sections were stained with an anti-smooth muscle actin antibody (Sigma) or Verhoeff's van Gieson stain (EVG) to visualise elastic lamina. Pulmonary vascular remodelling (muscularisation of small intrapulmonary arteries) was determined by counting all muscularised vessels with a diameter smaller than 50 μm in each lung section, and expressed as a % of all (muscularised + non-muscularised) vessels. In rats vascular remodelling was determined as evaluated as the proportion of peripheral vessels (<100 μm in diameter) vessels with double elastic lamina visualised with elastic van Gieson staining (>75% of the circumference as fully muscularised, 25–75% as partly muscularised) to total vessels counted. Counting was performed by observers blinded to treatment.

**Real-Time quantitative PCR.** Tissues were harvested and snap-frozen in liquid nitrogen. Approximately 20 mg of tissue was homogenized in a Mixer Mill MM 301 (Retsch GmbH, Haan, Germany) using tungsten carbide beads (Qiagen, Hilden, Germany). Total RNA was isolated from the lysate with the Invisorb Spin Tissue RNA Mini Kit (Invitek, Berlin, Germany). 100 ng total RNA was used for quantitative RT-PCR with the following amplicon sets: mCLIC4, forward ACAGCGAAGTCAAGACGGATG, reverse GACTCTGGGTGCTTTGGTGAA, probe TCGAAGAAGTCTTGTGCCCACCCAAGTA; mActin, forward GTTTGAGACCTTCAACACCCCA, reverse GACCAGAGGCATACAGGGACA, probe CCATGTACGTAGCCATCCAGGCTGTG (BioTez GmBH, Berlin, Germany). The reactions were carried out using an ABI PRISM 7700 Sequence Detector (Sequence Detection System v1.6.3, ABI Life Technologies), or a StepOnePlus Real Time PCR Sytem (ABI Life Technologies) with primers and probes at a concentration of 300 and 100 nmol/l respectively. For relative quantification, the data were analysed using the comparative Ct method.

## SUPPLEMENTAL REFERENCE

1. Vizcaino JA, Csordas A, Del-Toro N, Dianes JA, Griss J, Lavidas I, Mayer G, Perez-Riverol Y, Reisinger F, Ternent T, Xu QW, Wang R, Hermjakob H. 2016 update of the pride database and its related tools. *Nucleic acids research*. 2016;44:11033
2. Zhao Y, Gaidarov I, Keen JH. Phosphoinositide 3-kinase c2alpha links clathrin to microtubule-dependent movement. *J Biol Chem*. 2007;282:1249-1256
3. Jerabek-Willemsen M, Wienken CJ, Braun D, Baaske P, Duhr S. Molecular interaction studies using microscale thermophoresis. *Assay and drug development technologies*. 2011;9:342-353
4. Seidel SA, Dijkman PM, Lea WA, van den Bogaart G, Jerabek-Willemsen M, Lazic A, Joseph JS, Srinivasan P, Baaske P, Simeonov A, Katritch I, Melo FA, Ladbury JE, Schreiber G, Watts A, Braun D, Duhr S. Microscale thermophoresis quantifies biomolecular interactions under previously challenging conditions. *Methods*. 2013;59:301-315

**SUPPLEMENTAL TABLES**

**Online Table I.  Patient and healthy volunteer data**

Data presented as median (range)

| | | Control (n=6) | IPAH (n=6) |
|---|---|---|---|
| Males/Females | | 1/6 | 0/6 |
| Age (years) | | 27.0 (24.0 to 33.0) | 42.7 (27.0 to 67.0) |
| Time from diagnosis (months) | | - | 29.9 (12.0 to 60.0) |
| mPAP (mm Hg) | | - | 62.8 (50.0 to 71.0) |
| 6MWD (m) | | - | 366.4 (300 to 438) |
| WHO class | 1 | - | 0 |
| | 2 | - | 1 |
| | 3 | - | 4 |
| | 4 | - | 1 |
| Warfarin | | - | 4 |
| Treatment Naïve | | - | 1 |
| Calcium antagonist | | - | 0 |
| ER Antagonists | | - | 3 |
| PDE5 inhibitors | | - | 4 |
| Prostanoids | | - | 1 |
| Statins | | - | 0 |

**Online Table II. CLIC4-interacting proteins in HPAECs.** Proteins in bold were detected with large differences between Adcontrols and AdCLIC4 (at least 5 peptides in AdCLIC4 but none in AdControl). The remaining proteins were detected with 2-4 peptides in AdCLIC4 and none in AdControl.

| Protein | NP code |
|---|---|
| 60S ribosomal protein L3 | NP_000958.1 |
| acetyl-CoA carboxylase 1 | NP_942131.1 |
| actin-related protein 2/3 complex | NP_005711.1 |
| **ADP-ribosylation factor-like protein 6-interacting protein 4** | **NP_061164.2** |
| alpha-2-macroglobulin | NP_000005.2 |
| ammonium transporter Rh type A | NP_000315.2 |
| ankyrin repeat domain-containing protein 12 | NP_001190985.1 |
| **Arf GTPase-activating protein GIT1** | **NP_001078923.1** |
| **Arf GTPase-activating protein GIT2** | **NP_001128686.1** |
| ATP-dependent RNA helicase A | NP_001348.2 |
| ATP-dependent RNA helicase DDX1 | NP_004930.1 |
| ATP-dependent RNA helicase DDX18 | NP_006764.3 |
| ATP-dependent RNA helicase DDX3Y | NP_004651.2 |
| autophagy-related protein 2 homolog B | NP_060506.5 |
| **basement membrane-specific heparin sulphate proteoglycan** | **NP_005520.4** |
| BCL-6 corepressor | NP_060215.4 |
| caprin-1 | NP_005889.3 |
| centrosomal protein of 128 kDa | NP_689659.2 |
| chloride intracellular channel protein 1 | NP_001279.2 |
| chromosome-associated kinesin KIF4A | NP_036442.3 |
| **clathrin heavy chain 1** | **NP_004850.1** |
| coiled-coil domain-containing protein 150 | NP_001074008.1 |
| coiled-coil domain-containing protein 87 | NP_060689.2 |
| cytoskeleton-associated protein 2 | NP_060674.3 |
| dedicator of cytokinesis protein 4 | NP_055520.3 |
| desmoplakin | NP_004406.2 |
| DNA topoisomerase 2-binding protein 1 | NP_008958.2 |
| dynein heavy chain 14, axonemal | NP_001364.1 |
| dynein heavy chain 5, axonemal | NP_001360.1 |
| ETS translocation variant 3-like protein | NP_001004341.1 |
| fatty acid desaturase 1 | NP_037534.3 |
| FCH and double SH3 domains protein 1 | NP_258260.1 |
| fibronectin | NP_997639.1 |
| filamin-A | NP_001447.2 |
| hemoglobin | NP_000175.1 |
| heterogeneous nuclear ribonucleoprotein A/B | NP_004490.2 |
| heterogeneous nuclear ribonucleoprotein A1 | NP_002127.1 |
| heterogeneous nuclear ribonucleoprotein A3 | NP_919223.1 |
| heterogeneous nuclear ribonucleoprotein H3 | NP_067676.2 |
| heterogeneous nuclear ribonucleoprotein Q | NP_001153147.1 |

| | |
|---|---|
| heterogeneous nuclear ribonucleoprotein R | NP_005817.1 |
| histone acetyltransferase KAT7 | NP_008998.1 |
| histone H2B type 1-C/E/F/G/I | NP_003516.1 |
| histone H3.1 | NP_003520.1 |
| histone-lysine N-methyltransferase MLL | NP_005924.2 |
| inactive ubiquitin carboxyl-terminal hydrolase 54 | NP_689799.3 |
| interleukin enhancer-binding factor 2 (ILF2) | NP_004506.2 |
| interleukin enhancer-binding factor 3 (ILF3) | NP_036350.2 |
| katanin p60 ATPase-containing | NP_112593.2 |
| KN motif and ankyrin repeat domain-containing protein 2 | NP_056308.3 |
| lysosomal-trafficking regulator | NP_000072.2 |
| MICAL C-terminal-like protein | NP_116256.2 |
| microtubule-actin cross-linking factor 1 | NP_036222.3 |
| myosin-9 | NP_002464.1 |
| nesprin-1 | NP_892006.3 |
| nesprin-2 | NP_055995.4 |
| nestin | NP_006608.1 |
| neuroblast differentiation-associated protein AHNAK | NP_001611.1 |
| nuclear factor NF-kappa-B p65 | NP_001138610.1 |
| nuclear factor NF-kappa-B p105 | NP_003989.2 |
| peroxisome biogenesis factor 6 | NP_000278.3 |
| plectin | NP_958781.1 |
| polyadenylate-binding protein 3 | NP_112241.2 |
| protein unc-13 homolog C | NP_001074003.1 |
| RAD51-associated protein 2 | NP_001092688.1 |
| ranBP2-like and GRIP domain-containing protein 3 | NP_001137485.1 |
| replication protein A 70 kDa DNA-binding | NP_002936.1 |
| RNA-binding protein 14 | NP_006319.1 |
| separin | NP_036423.4 |
| serine/threonine-protein kinase SMG1 | NP_055907.3 |
| signal peptide, CUB and EGF-like domain-containing protein 3 | NP_689966.2 |
| splicing factor, proline- and glutamine-rich | NP_005057.1 |
| synaptotagmin-like protein 4 | NP_542775.2 |
| syntaxin-binding protein 1 | NP_001027392.1 |
| talin-1 | NP_006280.3 |
| TATA-binding protein-associated factor 2N | NP_631961.1 |
| teneurin-3 | NP_001073946.1 |
| titin | NP_596870.2 |
| trafficking kinesin-binding protein 1 | NP_001036111.1 |
| transcription factor 20 | NP_005641.1 |
| ubiquitin conjugation factor E4 A | NP_004779.2 |
| uncharacterized protein KIAA1109 | NP_056127.2 |
| WD repeat and FYVE domain-containing protein 3 | NP_055806.2 |
| zinc finger protein 598 | NP_835461.1 |

**Online Table III.** Selected differentially expressed proteins in CLIC4 over-expressing HPAECs involved in the regulation of the endocytotic pathway, lysosomal degradation trafficking, NFκB and TNF-α signalling, leucocyte trafficking and others.

| **Protein** | **NP number** | **Gene Symbol** | **Molecular Weight (kDa)** | **Peptide count** | **Fold difference** | **P-value** |
|---|---|---|---|---|---|---|
| Endocytotic pathway | | | | | | |
| teneurin-3 | NP_001073946.1 | TENM3 | 300.9 | 2 | +21.43 | 0.0222 |
| BTB/POZ domain-containing protein 6 | NP_150374.2 | BTBD6 | 53.4 | 2 | +5.45 | 0.0002 |
| golgin subfamily A member 2 | NP_004477.3 | GOLGA2 | 113.1 | 6 | +3.38 | 0.0452 |
| disintegrin and metalloproteinase domain-containing protein 33 | NP_079496.1 | ADAM33 | 87.7 | 4 | +3.33 | 0.0003 |
| plakophilin-3 | NP_009114.1 | PKP3 | 87.1 | 2 | +2.22 | 0.0114 |
| [F-actin]-monooxygenase MICAL1 | NP_001152763.1 | MICAL1 | 108.6 | 2 | +1.93 | 0.0080 |
| paralemmin 3 | NP_001138500.1 | PALM3 | 71.7 | 2 | +1.66 | 0.0009 |
| intersectin-2 | NP_006268.2 | ITSN2 | 193.5 | 2 | 1.57 | 0.0094 |
| Protein SGT1 homolog | NP_006695.1 | SUGT1 | 37.8 | 2 | -1.73 | 0.0005 |
| C-type mannose receptor 2 | NP_006030.2 | MRC2 | 166.7 | 2 | -1.65 | 0.0325 |
| F-box only protein 3 | NP_208385.1 | FBXO3 | 47.5 | 2 | -1.63 | 0.0156 |
| Prolow-density lipoprotein receptor-related protein 1 | NP_002323.2 | LRP1 | 504.6 | 4 | -1.59 | 0.0113 |
| protein GPR107 | NP_066011.2 | GPR107 | 62.0 | 2 | -1.58 | 0.0057 |

| | | | | | | |
|---|---|---|---|---|---|---|
| <u>Lysosomal pathway</u> | | | | | | |
| E3 ubiquitin-protein ligase RNF6 | NP_898865.1 | RNF6 | 78.1 | 4 | +2.5 | 0.0005 |
| synaptophysin | NP_003170.1 | SYP | 33.8 | 2 | +1.94 | 0.0064 |
| TRAF3-interacting protein 1 | NP_056465.2 | TRAF3IP1 | 78.6 | 2 | +1.81 | 0.0175 |
| Dynein regulatory complex subunit 7 | NP_115645.4 | DRC7 | 103.5 | 7 | +1.77 | 0.0247 |
| ubiquitin carboxyl-terminal hydrolase 25 | NP_037528.3 | USP25 | 122.2 | 3 | +1.74 | 0.0216 |
| ubiquitin thioesterase ZRANB1 | NP_060050.2 | ZRANB1 | 81.0 | 2 | +1.73 | 0.0376 |
| E3 ubiquitin-protein ligase TRAF7 | NP_115647.2 | TRAF7 | 74.6 | 2 | -2.23 | 0.0262 |
| E3 ubiquitin-protein ligase UBR2 | NP_056070.1 | UBR2 | 200.5 | 2 | -1.77 | 0.0013 |
| coiled-coil and C2 domain containing 1A | NP_060191.3 | CC2D1A | 104.1 | 4 | -1.64 | 0.0237 |
| <u>NFkB & TNF-α signalling</u> | | | | | | |
| AMP deaminase 3 | NP_001020561.1 | AMPD3 | 89.5 | 2 | +16.68 | 0.0005 |
| chondroitin sulfate proteoglycan 4 | NP_001888.2 | CSPG4 | 250.5 | 2 | +12.87 | 0.0003 |
| mitogen-activated protein kinase kinase kinase 4 | NP_005913.2 | MAP3K4 | 181.7 | 2 | +6.68 | 0.0001 |
| insulin receptor substrate 1 | NP_005535.1 | IRS1 | 131.6 | 2 | +4.14 | 0.0007 |
| protein arginine N-methyltransferase 5 | NP_006100.2 | PRMT5 | 72.7 | 2 | +2.55 | 0.0003 |
| DNA topoisomerase 1 | NP_003277.1 | TOP1 | 90.7 | 4 | +2.4 | 0.0007 |
| paternally expressed 3 | NP_001139657.1 | PEG3 | 166.0 | 4 | +1.72 | 0.0004 |
| myosin light chain kinase 3 | NP_872299.2 | MYLK3 | 88.4 | 4 | -1.99 | 0.0257 |

| | | | | | | |
|---|---|---|---|---|---|---|
| progressive ankylosis protein homolog | NP_473368.1 | ANKH | 54.2 | 2 | -1.98 | 0.0039 |
| BRCA1-associated RING domain protein 1 | NP_000456.2 | BARD1 | 86.6 | 2 | -1.72 | 0.0100 |
| thrombospondin-4 | NP_003239.2 | THBS4 | 105.9 | 2 | -1.68 | 0.0424 |
| filaggrin | NP_002007.1 | FLG | 435.2 | 4 | -1.64 | 0.0030 |
| interferon regulatory factor 1 | NP_002189.1 | IRF1 | 36.5 | 2 | -1.57 | 0.0016 |
| kelch domain-containing protein 10 | NP_055812.1 | KLHDC10 | 49.1 | 4 | -1.56 | 0.0073 |
| Leucocyte trafficking | | | | | | |
| fermitin family homolog 3 long form | NP_848537.1 | FERMT3 | 76.0 | 4 | +3.81 | 0.0003 |
| vitamin D-binding protein | NP_000574.2 | GC | 52.9 | 2 | -1.56 | 0.0188 |
| integrin alpha-M isoform 2 | NP_000623.2 | ITGAM | 127.2 | 2 | -1.6 | 0.0350 |
| sphingomyelin phosphodiesterase 2 | NP_003071.2 | SMPD2 | 47.6 | 2 | -1.63 | 0.0246 |
| tyrosine-protein kinase Tec | NP_003206.2 | TEC | 73.6 | 2 | -1.64 | 0.0083 |
| Perlecan/heparan sulfate protein | NP_005520.4 | HSPG2 | 468.8 | 2 | -1.89 | 0.0034 |
| protein tyrosine phosphatase, receptor type, J | NP_002834.3 | PTPRJ | 145.9 | 2 | -2.09 | 0.0004 |
| protein-tyrosine kinase 2-beta | NP_004094.3 | PTK2B | 115.9 | 2 | -2.1 | 0.0255 |
| Cell structure, movement & transport | | | | | | |
| Potassium/sodium hyperpolarization-activated cyclic nucleotide-gated channel 2 | NP_001185.3 | HCN2 | 96.9 | 2 | +34.41 | 0.0005 |
| formin-2 | NP_064450.3 | FMN2 | 180.1 | 2 | +22.51 | 0.0002 |
| alanine and arginine-rich domain-containing protein | NP_001020528.1 | AARD | 17.6 | 2 | +11.82 | 0.0006 |

| | | | | | | |
|---|---|---|---|---|---|---|
| C-terminal-binding protein 1 | NP_001319.1 | CTBP1 | 47.5 | 2 | +6.49 | 0.0000 |
| group 3 secretory phospholipase A2 | NP_056530.2 | PLA2G3 | 57.2 | 2 | +5.9 | 0.0017 |
| sodium/nucleoside cotransporter 1 | NP_004204.3 | SLC28A1 | 71.6 | 2 | +3.52 | 0.0045 |
| collagen alpha-1(XIV) chain | NP_066933.1 | COL14A1 | 193.5 | 6 | +3.17 | 0.0267 |
| Capping protein, Arp2/3 and myosin-I linker protein 3 | NP_612369.3 | CARMIL3 | 150.2 | 2 | +3.04 | 0.0001 |
| sushi repeat-containing protein SRPX2 | NP_055282.1 | SRPX2 | 53.0 | 2 | +2.27 | 0.0183 |
| beta-tubulin cofactor D | NP_005984.3 | TBCD | 132.6 | 2 | +2.23 | 0.0149 |
| BR serine/threonine-protein kinase 1 | NP_115806.1 | BRSK1 | 85.1 | 2 | +1.85 | 0.0278 |
| abnormal spindle-like microcephaly-associated protein | NP_060606.3 | ASPM | 409.8 | 7 | +1.67 | 0.0009 |
| filamin-C | NP_001449.3 | FLNC | 291.0 | 3 | +1.62 | 0.0422 |
| dedicator of cytokinesis protein 3 | NP_004938.1 | DOCK3 | 233.1 | 2 | +1.61 | 0.0328 |
| Protein phosphatase 1 regulatory subunit 26 | NP_055626.3 | PPP1R26 | 127.4 | 3 | +1.52 | 0.0043 |
| partitioning defective 3 homolog B | NP_995585.2 | PARD3B | 121.2 | 2 | -3.1 | 0.0016 |
| Mast/stem cell growth factor receptor Kit | NP_000213.1 | KIT | 109.9 | 2 | -3.08 | 0.0026 |
| dihydropyrimidinase-related protein 2 | NP_001377.1 | DPYSL2 | 62.3 | 2 | -2.51 | 0.0302 |
| nuclear migration protein nudC | NP_006591.1 | NUDC | 38.2 | 2 | -2.32 | 0.0008 |
| cytoplasmic dynein 1 heavy chain 1 | NP_001367.2 | DYNC1H1 | 532.4 | 7 | -2.18 | 0.0216 |
| kinesin family member 3A | NP_008985.3 | KIF3A | 80.0 | 3 | -2.06 | 0.0337 |
| TRIO and F-actin-binding protein | NP_008963.3 | TRIOBP | 74.0 | 2 | -2.01 | 0.0485 |

| | | | | | | |
|---|---|---|---|---|---|---|
| Spectrin alpha chain, non-erythrocytic 1 | NP_001123910.1 | SPTAN1 | 285.1 | 2 | -1.98 | 0.0332 |
| plastin-3 | NP_005023.2 | PLS3 | 70.8 | 2 | -1.97 | 0.0039 |
| tetratricopeptide repeat protein 25 | NP_113609.1 | TTC25 | 76.7 | 2 | -1.92 | 0.0002 |
| Neutral amino acid transporter B(0) | NP_005619.1 | SLC1A5 | 56.6 | 2 | -1.74 | 0.0219 |
| hornerin | NP_001009931.1 | HRNR | 282.4 | 4 | -1.69 | 0.0192 |
| 28S ribosomal protein S27, mitochondrial | NP_055899.2 | MRPS27 | 47.6 | 2 | -1.68 | 0.0048 |
| synaptopodin 2-like protein | NP_079151.2 | SYNPO2L | 79.1 | 5 | -1.66 | 0.0246 |
| WD repeat and HMG-box DNA-binding protein 1 | NP_009017.1 | WDHD1 | 126.0 | 2 | -1.64 | 0.0002 |
| acetylcholine receptor subunit epsilon | NP_000071.1 | CHRNE | 54.7 | 3 | -1.63 | 0.0236 |
| CAP-Gly domain-containing linker protein 1 | NP_937883.1 | CLIP1 | 156.8 | 5 | -1.63 | 0.0082 |
| protocadherin gamma-B1 isoform 1 | NP_061745.1 | PCDHGB1 | 100.4 | 2 | -1.62 | 0.0253 |
| armadillo repeat-containing protein 4 | NP_060546.2 | ARMC4 | 115.7 | 3 | -1.62 | 0.0087 |
| matrix metalloproteinase-14 | NP_004986.1 | MMP14 | 65.9 | 2 | -1.59 | 0.0158 |
| Others | | | | | | |
| brain-enriched guanylate kinase-associated protein | NP_065887.1 | BEGAIN | 64.8 | 2 | +14.14 | 0.0003 |
| dual oxidase 1 | NP_787954.1 | DUOX1 | 177.2 | 2 | +14.12 | 0.0005 |
| Fanconi-associated nuclease 1 | NP_055782.3 | FAN1 | 114.2 | 2 | +12.03 | 0.0026 |
| peregrin | NP_001003694.1 | BRPF1 | 138.2 | 2 | +8.98 | 0.0004 |
| g patch domain-containing protein 8 | NP_001002909.1 | GPATCH8 | 164.2 | 2 | +6.92 | 0.0011 |

| | | | | | | |
|---|---|---|---|---|---|---|
| PR domain zinc finger protein 13 | NP_067633.2 | PRDM13 | 74.0 | 2 | +6.8 | 0.0001 |
| galactose-3-O-sulfotransferase 2 | NP_071417.2 | GAL3ST2 | 46.1 | 3 | +3.25 | 0.0004 |
| histidyl-tRNA synthetase, cytoplasmic | NP_002100.2 | HARS | 57.4 | 2 | +2.27 | 0.0168 |
| immunoglobulin-like domain containing receptor 2 | NP_955383.1 | ILDR2 | 71.2 | 3 | +2.15 | 0.0046 |
| MBT domain-containing protein 1 | NP_060113.2 | MBTD1 | 70.5 | 2 | +2.1 | 0.0000 |
| zinc finger protein 516 | NP_055458.1 | ZNF516 | 124.3 | 2 | +2.03 | 0.0435 |
| immunoglobulin-like and fibronectin type III | NP_001158058.1 | IGFN1 | 383.8 | 6 | +1.89 | 0.0439 |
| lamin-A/C isoform 1 | NP_733821.1 | LMNA | 74.1 | 2 | +1.76 | 0.0317 |
| ribosomal protein L6 | NP_000961.2 | RPL6 | 32.7 | 4 | +1.68 | 0.0115 |
| C2 calcium-dependent domain-containing protein | NP_001129735.1 | C2CD4C | 44.6 | 2 | +1.68 | 0.0353 |
| MAX gene-associated protein | NP_001074010.2 | MGA | 315.2 | 2 | +1.67 | 0.0429 |
| zinc finger and BTB domain-containing protein 4 | NP_065950.2 | ZBTB4 | 105.1 | 2 | +1.64 | 0.0008 |
| Membrane-associated phosphatidylinositol transfer protein 2 | NP_065896.1 | PITPNM2 | 148.9 | 2 | +1.63 | 0.0075 |
| retinoic acid induced 16 | NP_073586.5 | FAM160B2 | 82.3 | 2 | +1.52 | 0.0034 |
| Striated muscle preferentially expressed protein | NP_005867.3 | SPEG | 354.3 | 5 | +1.52 | 0.0189 |
| replication protein A1 | NP_002936.1 | RPA1 | 68.1 | 2 | -4.61 | 0.0278 |
| progesterone receptor membrane component 2 | NP_006311.2 | PGRMC2 | 26.2 | 2 | -3.42 | 0.0204 |
| GC-rich sequence DNA-binding factor | NP_003194.3 | GCFC2 | 89.4 | 2 | -2.97 | 0.0012 |
| alpha-2-HS-glycoprotein | NP_001613.2 | AHSG | 39.3 | 2 | -2.92 | 0.0287 |

| | | | | | | |
|---|---|---|---|---|---|---|
| neuroligin-2 | NP_065846.1 | NLGN2 | 90.8 | 2 | -2.48 | 0.0249 |
| phosphoribosylformylglycinamidine synthase | NP_036525.1 | PFAS | 144.7 | 2 | -2.3 | 0.0462 |
| amiloride-sensitive cation channel 4 | NP_061144.3 | ASIC4 | 72.2 | 2 | -2.2 | 0.0249 |
| heterogeneous nuclear ribonucleoprotein H2 | NP_062543.1 | HNRNPH2 | 49.3 | 2 | -2.06 | 0.0469 |
| MPN domain-containing protein | NP_116257.2 | MPND | 50.7 | 2 | -2.05 | 0.0007 |
| phosphatase and actin regulator 2 | NP_001093634.1 | PHACTR2 | 70.7 | 2 | -2.02 | 0.0018 |
| DNA polymerase iota | NP_009126.2 | POLI | 83.0 | 5 | -2.02 | 0.0000 |
| protein Wiz | NP_067064.2 | WIZ | 85.4 | 3 | -2.01 | 0.0024 |
| lysine-specific demethylase 4A | NP_055478.2 | KDM4A | 120.7 | 2 | -1.98 | 0.0021 |
| prostaglandin E2 receptor EP1 | NP_000946.2 | PTGER1 | 41.8 | 2 | -1.97 | 0.0219 |
| cytochrome P450 2C9 | NP_000762.2 | CYP2C9 | 55.6 | 4 | -1.92 | 0.0036 |
| tRNA wybutosine-synthesizing protein 2 | NP_060426.2 | TRMT12 | 50.2 | 2 | -1.9 | 0.0452 |
| heparan-sulfate 6-O-sulfotransferase 1 | NP_004798.3 | HS6ST1 | 48.2 | 2 | -1.89 | 0.0283 |
| polycomb protein SCMH1 | NP_001165690.1 | SCMH1 | 72.1 | 2 | -1.86 | 0.0071 |
| dapper homolog 3 | NP_659493.2 | DACT3 | 64.9 | 3 | -1.86 | 0.0118 |
| Histone-lysine N-methyltransferase KMT5B | NP_060105.3 | KMT5B | 99.2 | 2 | -1.85 | 0.0086 |
| pericentrin | NP_006022.3 | PCNT | 378.0 | 5 | -1.8 | 0.0005 |
| transcription initiation factor TFIID | NP_722516.1 | TAF1L | 207.3 | 3 | -1.77 | 0.0031 |
| synaptonemal complex protein 2 | NP_055073.2 | SYCP2 | 175.6 | 2 | -1.76 | 0.0044 |

| | | | | | | |
|---|---|---|---|---|---|---|
| splicing factor 3A subunit 3 | NP_006793.1 | SF3A3 | 58.8 | 2 | -1.74 | 0.0192 |
| potassium channel subfamily K member 10 | NP_066984.1 | KCNK10 | 59.8 | 2 | -1.74 | 0.0013 |
| Forkhead-associated domain-containing protein 1 | NP_443161.1 | FHAD1 | 161.9 | 2 | -1.73 | 0.0061 |
| retinal-specific ATP-binding cassette transporter | NP_000341.2 | ABCA4 | 255.9 | 2 | -1.73 | 0.0344 |
| zinc finger family member 674 | NP_001034980.1 | ZNF674 | 67.2 | 2 | -1.7 | 0.0176 |
| glucosamine--fructose-6-phosphate aminotransferase | NP_002047.2 | GFPT1 | 76.8 | 2 | -1.7 | 0.0007 |
| golgi apparatus protein 1 | NP_036333.2 | GLG1 | 137.2 | 2 | -1.7 | 0.0022 |
| peroxisomal acyl-coenzyme A oxidase 3 | NP_003492.2 | ACOX3 | 77.6 | 2 | -1.69 | 0.0138 |
| retinitis pigmentosa 1-like 1 protein | NP_849188.4 | RP1L1 | 252.3 | 2 | -1.68 | 0.0075 |
| amine oxidase | NP_000889.3 | MAOB | 58.8 | 2 | -1.67 | 0.0169 |
| sperm-specific antigen 2 | NP_006742.2 | SSFA2 | 137.9 | 2 | -1.67 | 0.0157 |
| eukaryotic initiation factor 4A-I | NP_001407.1 | EIF4A1 | 46.2 | 2 | -1.65 | 0.0435 |
| Meiosis regulator and mRNA stability factor 1 | NP_055462.2 | MARF1 | 192.9 | 2 | -1.65 | 0.0015 |
| ectonucleotide pyrophosphatase/phosphodiesterase | NP_006199.2 | ENPP1 | 104.9 | 2 | -1.64 | 0.0001 |
| A-kinase anchor protein 11 | NP_057332.1 | AKAP11 | 210.5 | 2 | -1.64 | 0.0301 |
| zinc finger protein 764 | NP_219363.2 | ZNF764 | 44.9 | 4 | -1.64 | 0.0263 |
| cytochrome c oxidase assembly protein COX11, mitochondrial | NP_004366.1 | COX11 | 31.4 | 2 | -1.61 | 0.0158 |
| protein KRBA1 | NP_115923.2 | KRBA1 | 107.5 | 2 | -1.61 | 0.0119 |
| multiple epidermal growth factor-like domains protein | NP_001400.3 | MEGF6 | 161.2 | 2 | -1.61 | 0.0046 |

| | | | | | | |
|---|---|---|---|---|---|---|
| histone acetyltransferase KAT6B | NP_036462.2 | KAT6B | 231.4 | 3 | -1.61 | 0.0031 |
| alpha 3 type VI collagen | NP_476507.3 | COL6A3 | 278.2 | 3 | -1.61 | 0.0292 |
| low-density lipoprotein receptor-related protein | NP_004516.2 | LRP2 | 522.0 | 2 | -1.61 | 0.0226 |
| metabotropic glutamate receptor 2 | NP_001123535.1 | GRM2 | 27.5 | 2 | -1.59 | 0.0034 |
| ankyrin repeat domain 56 | NP_001025041.1 | SOWAHB | 85.7 | 5 | -1.59 | 0.0050 |
| AHNAK nucleoprotein 2 | NP_612429.2 | AHNAK2 | 616.6 | 2 | -1.58 | 0.0148 |
| protein bicaudal D homolog 2 | NP_056065.1 | BICD2 | 93.5 | 2 | -1.57 | 0.0122 |
| zinc finger C3H1 domain-containing protein | NP_659419.3 | ZFC3H1 | 226.4 | 6 | -1.57 | 0.0015 |
| N-acetylglutamate synthase, mitochondrial | NP_694551.1 | NAGS | 58.2 | 2 | -1.57 | 0.0106 |
| DNA-directed DNA polymerase beta | NP_002681.1 | POLB | 38.2 | 2 | -1.56 | 0.0009 |
| actin-related protein 8 | NP_075050.3 | ACTR8 | 70.5 | 2 | -1.56 | 0.0055 |
| zinc finger protein 33B | NP_008886.1 | ZNF33B | 90.7 | 4 | -1.56 | 0.0007 |
| platelet-activating factor acetylhydrolase | NP_005075.3 | PLA2G7 | 50.1 | 2 | -1.56 | 0.0305 |

**SUPPLEMENTAL FIGURES**

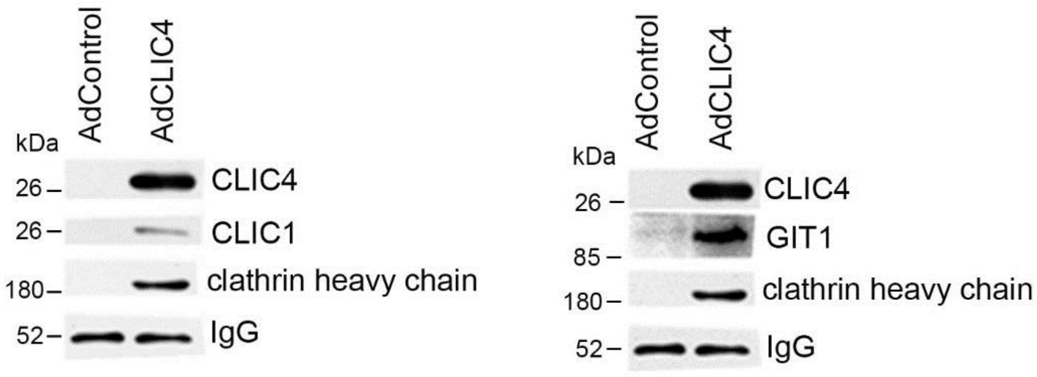


**Online Figure I. Clathrin heavy chain and GIT1 interact with CLIC4 in HPAECs**. Proteins associated with exogenously expressed HA-tagged CLIC4 were immunoprecipitated in HPAEC lysates with anti-HA antibody, resolved by electrophoresis and identified by western blotting analysis. Blots were probed with goat polyclonal anti-GIT1 antibody (sc-9657, Santa Cruz Biotechnology) and rabbit anti-clathrin heavy chain antibody (ab21679, Abcam).

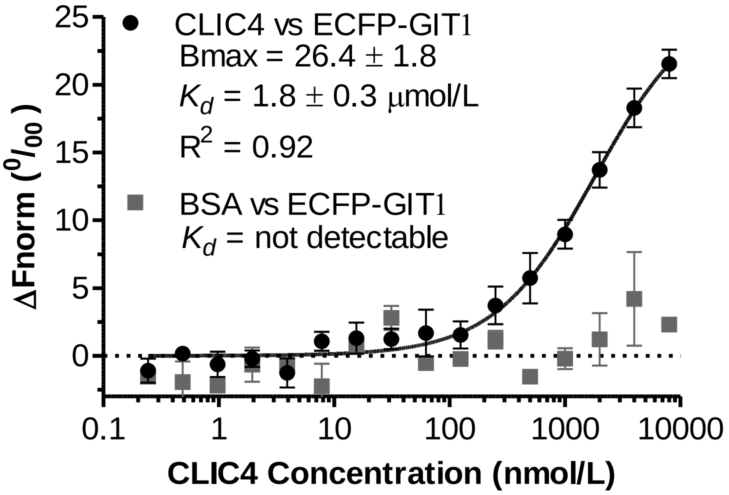


**Online Figure II. Microscale Thermophoresis (MST) binding curves using CLIC4 and ECFP-labelled GIT1**. BSA was used as a control to test for non-specific binding. Normalised fluorescence was calculated using NanoTemper Analysis 1.2.101 software and is plotted as a function of CLIC4 concentration (nmol/L). Estimated binding affinity was calculated by fitting a one-site binding curve to the data. The estimated Bmax, $K_d$ and $R^2$ values are indicated (*inset*) with SEM. Measurements were repeated at least three times for CLIC4 and twice for the BSA control.

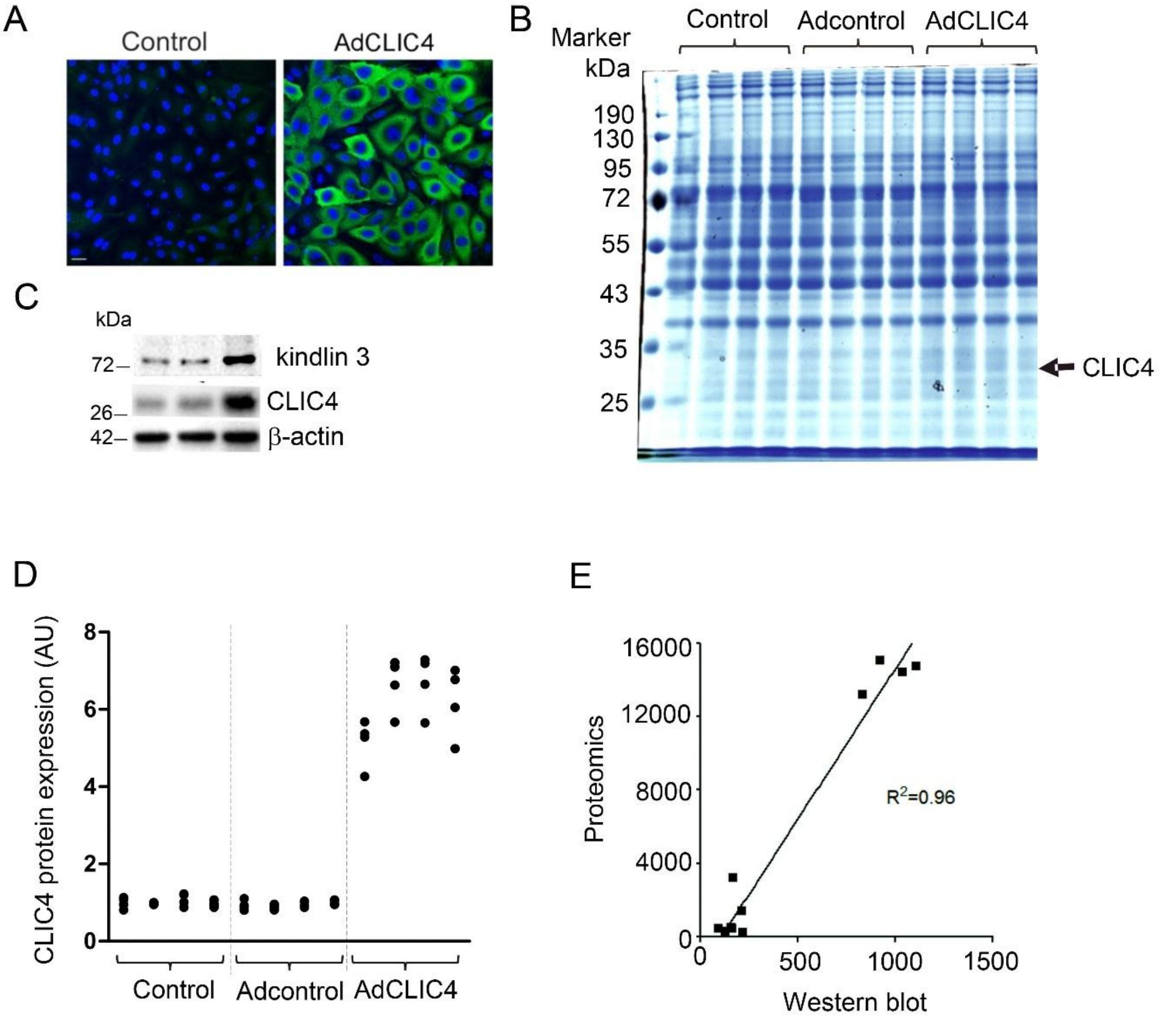


**Online Figure III. Determination of CLIC4 expression levels using immunohistochemistry, western blotting and proteomics.** (A) Representative images showing CLIC4 expression in the untreated (control) HPAECs and HPAECs infected with AdCLIC4 (24hr post-infection), as indicated. Nuclei are blue and CLIC4 is green. Bar=10 µm (B) SDS–PAGE separation of HPAEC lysates (10 µg protein/lane) from the untreated (Control) cells or cells infected with AdTet-off (AdControl) or AdCLIC4 (n=4/group); (C) western blots confirming elevated expression levels of kindlin 3 in CLIC4-overepressing cells; (D) Expression levels of 4 peptide ions derived from CLIC4 (DEFTNTCPSDK, EVEIAYSDVAK, YLTNAYSR, GVVFSVTTVDLK) in the proteomics analysis. Results indicate an average of 6-fold increase in the AdCLIC4 group compared to Adcontrol. (E) Measurements of relative CLIC4 protein levels for each sample determined by proteomics (sum of peptide ion intensity values) and densitometry of western blots (arbitrary units), indicating a strong correlation between the two parameters.

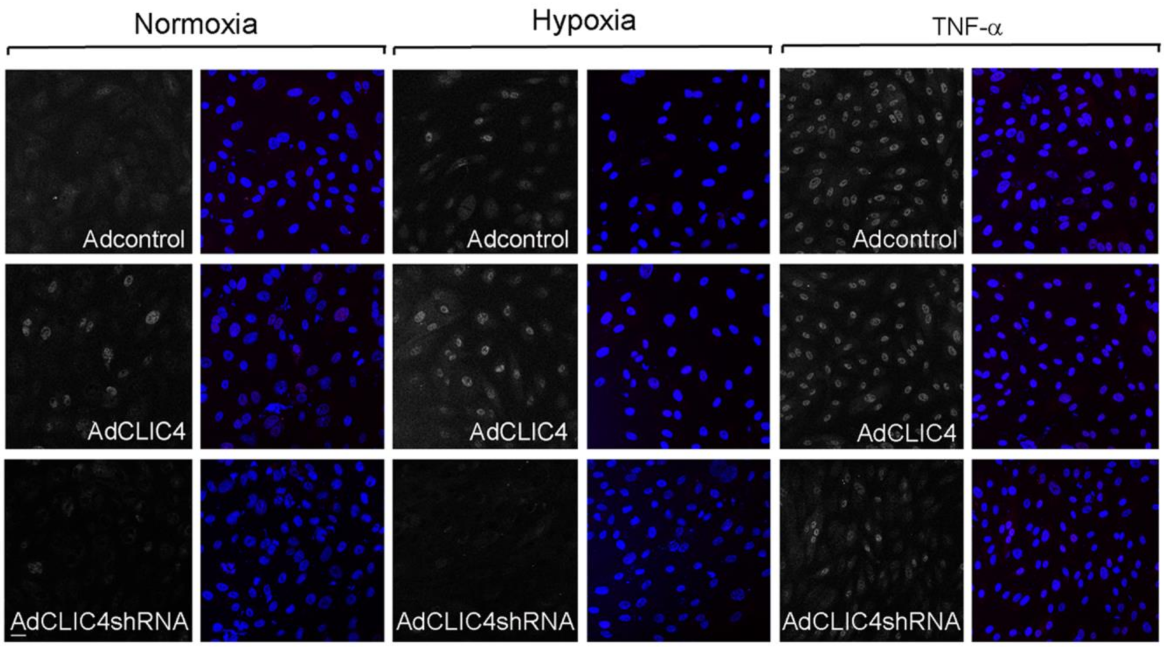


**Online Figure IV. Nuclear translocation of NFκB**. Nuclear translocation of NFκB induced by hypoxia or TNF-α (10 ng/mL, 24hr) in HPAECs overexpressing AdGFP (Adcontrol), AdCLIC4 or AdCLIC4shRNA, as indicated. Black-and-white images show localization of p65NFκB while corresponding colour images show merged nuclear (DAPI; blue) and NFκB staining (red). Bar=20 µm.

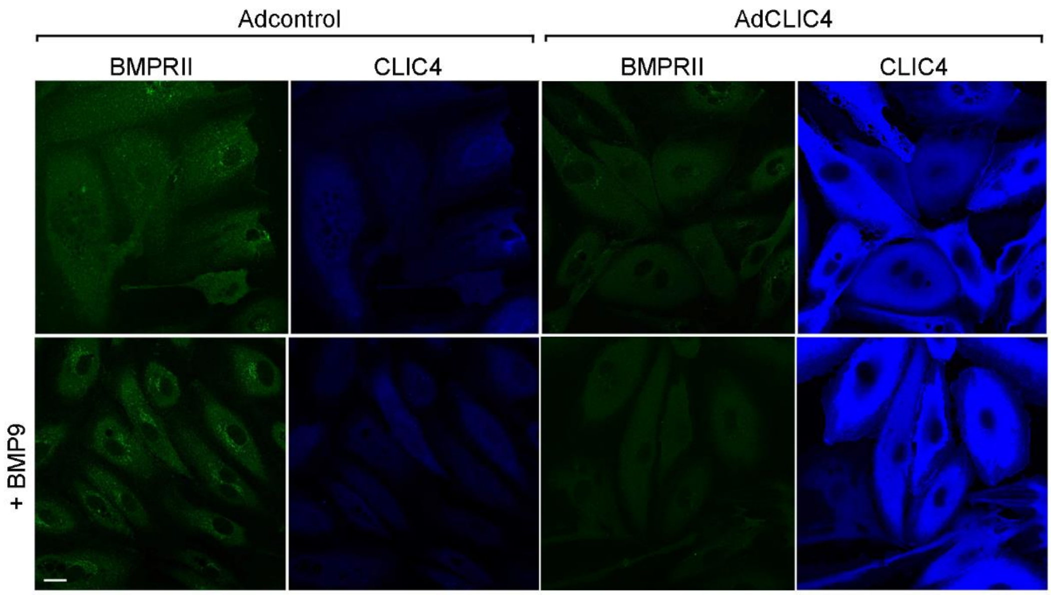


**Online Figure V**. **CLIC4 overexpression reduces protein levels of BMPRII.** Overexpression of CLIC4 reduces the levels of endogenous BMPRII in the untreated and BMP9-treated cells, as indicated. HPAECs were infected with control adenoviruses (Adcontrol) or AdCLIC4 (AdCLIC4) and were left untreated or were transfected with BMPRII-GFP and incubated for further 24hr. BMP9 (10 µg/L) was added to the cells 1hr before the end of experiment. In corresponding confocal images BMPRII is green and CLIC4 is blue. Bar=10 µm

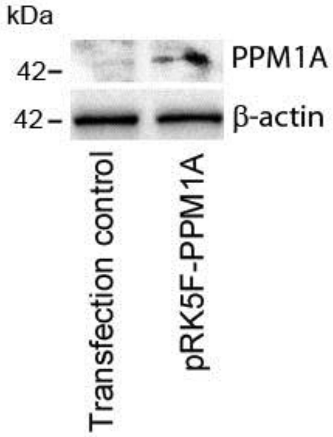


**Online Figure VI. PPM1A overexpression in HPAECs.** HPAECs were transfected with pRK5F-PPM1A. Increased expression levels of PPM1A were confirmed by western blotting.

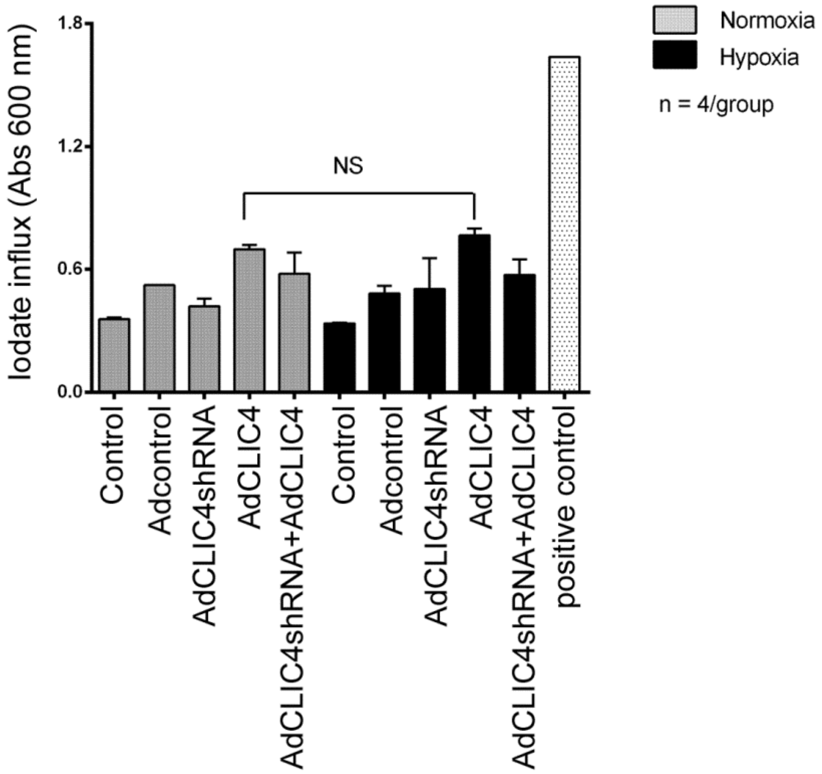


**Online Figure VII. Cell plasma membrane anion permeability.** Passive cellular iodide uptake was measured with the Chloride Channel Assay kit (Abcam). The cells were overexpressing AdCLIC4 or ADCLIC4shRNA in normoxic or in hypoxic conditions, as indicated.n=4.Data are presented as mean ±SEM. One-way ANOVA with Tukey's post-hoc test.

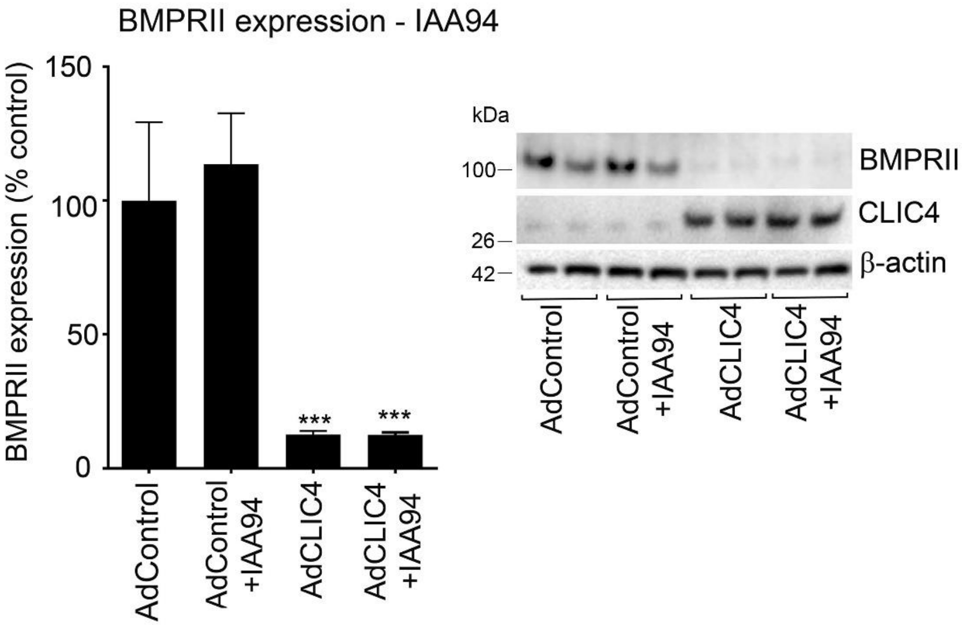


**Online Figure VIII. The effect of IAA-94 on BMPRII expression in CLIC4 overexpressing HPAECs.** Graph and representative western blots show protein levels of BMPRII in control (Adcontrol) and CLIC4-overexpressing (AdCLIC4) cells cultured with, or without chloride channel inhibitor, Indanyloxy acetic acid (IAA94, 100 µmol/L, 24hr). n=4. ***$P<0.001$, compared to Adcontrols; Data are presented as mean ±SEM. One-way ANOVA with Tukey's post-hoc test.

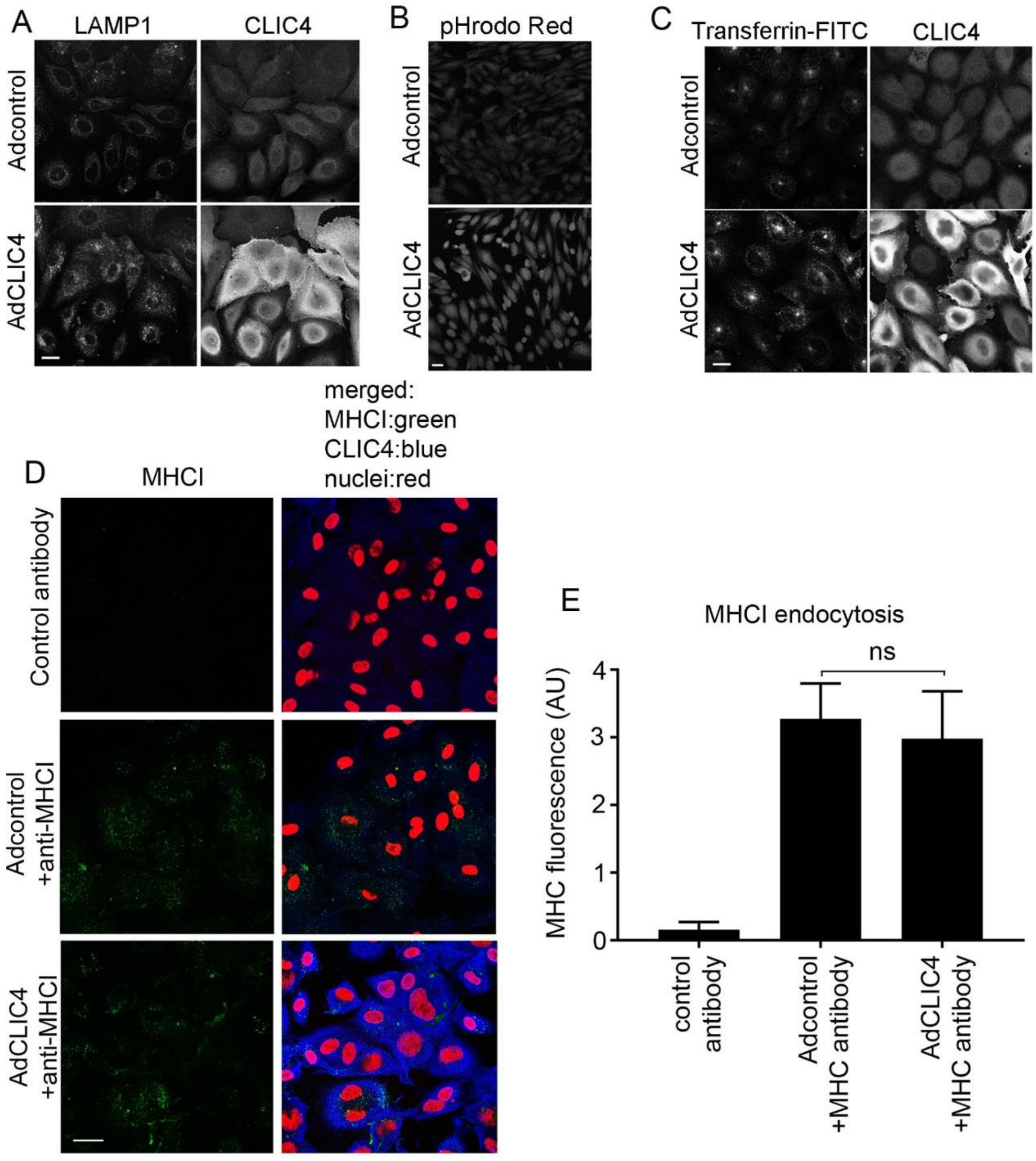


**Online Figure IX. CLIC4 increases the number and acidification of lysosomes and increases clathrin-mediated receptor internalization.** (A) Overexpression of CLIC4 increases intracellular levels of LAMP-1-positive vesicles. HPAECs were infected with Adcontrol or AdCLIC4 and LAMP-1 localization was analysed by immunofluorescence and confocal microscopy. (B) CLIC4 increases lysosomal acidification measured with fluorescent PH indicator pHrodo Red. (C) CLIC4 increases the uptake of Alexa488-transferrin in HPAECs. (D-E) Confocal images and graph show the levels of internalised, fluorescently-labelled MHCI; n=3; Student t-test. Bar=10 µm.

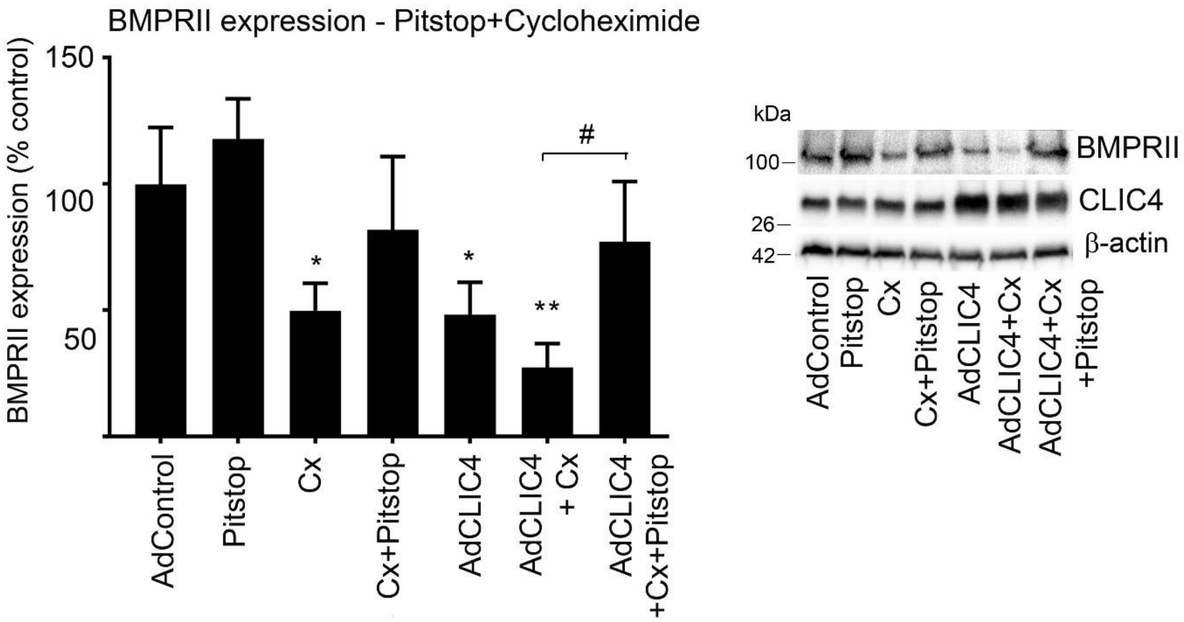


**Online Figure X. Pitstop2 restores BMPRII levels in CLIC4-overexpressing cells.** Control (Adcontrol) and AdCLIC4-overexpressing (AdCLIC4) cells were treated with 20 mg/L cycloheximide, with or without the endocytosis inhibitor Pitsop2 (20 µmol/L; ab120687, Abcam) or Pitstop2 negative control (20 µmol/L; ab120688, Abcam) for 2hr. Negative control compound was added to all cells except for cells cultured with Pitstop2. The cells were then lysed and changes in BMPRII expression were analysed by western blotting. Graph and corresponding, representative western blots show BMPRII expression changes in cells treated, as indicated. Data presented as mean±SEM; n=4, *P<0.05, **P<0.01, comparison with Adcontrol; #P<0.05, comparison, as indicated, one-way ANOVA with Tukey's post-hoc test.

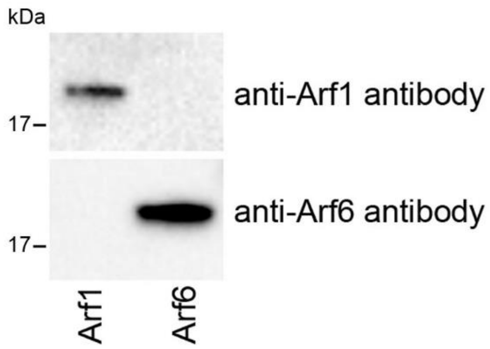


**Online Figure XI. Western blot images showing specificity of anti- Arf6 and anti-Arf1 antibodies.** Arf1 (Part#A1CA, Cytoskeleton Inc, 0.5 ng/lane) or Arf6 (Part#A6CA, Cytoskeleton Inc., 0.5 ng/lane) protein standards were probed with either Arf1 or Arf6 antibodies to determine specificity, as indicated. No cross-reactivity was observed.

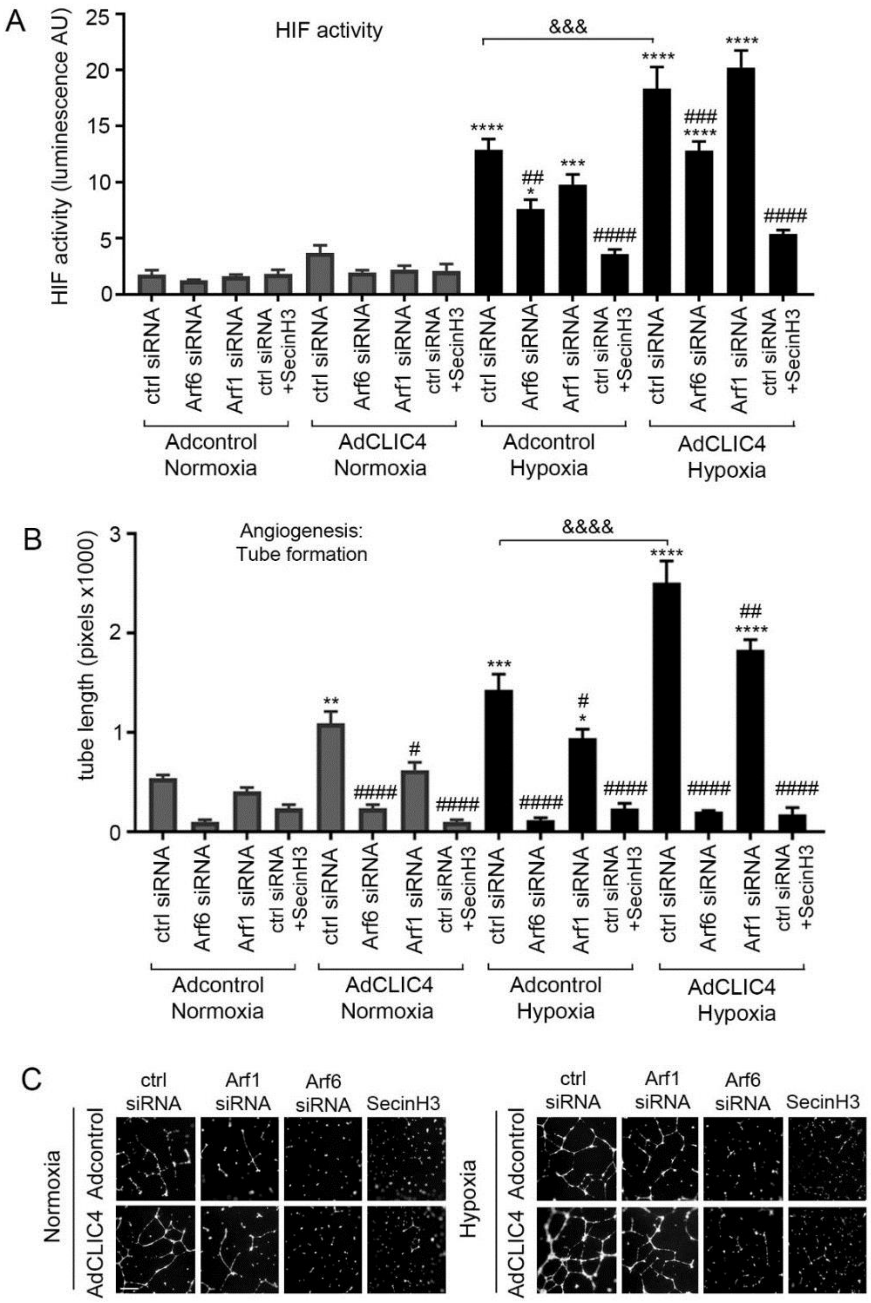


**Online Figure XII. The effect of SecinH3, Arf1 and Arf6 silencing on CLIC4-induced HIF activation and endothelial tube formation.** (A) HIF activation in U2OS cells transfected with silencing RNAs (control siRNA, Arf6 siRNA and Arf1 siRNA), with or without CLIC4 overexpression, with or without SecinH3 and cultured in normoxic or in hypoxic conditions, as indicated; luciferase reporter assay. (B) Tube formation in matrigel (total tube length) in cells treated, as indicated. (C) Representative images of tube formation in live cells stained with Cell Tracker Green; fluorescence microscopy. Bar=50 µm. *P<0.05, **P<0.001, ***P<0.001, comparisons with controls (control siRNA + Adcontrol in normoxia); #P<0.05, ##P<0.01; #P<0.05, ##P<0.01, ###P<0.001, comparisons with corresponding ctrl siRNA controls, &&&P<0.001, comparison, as indicated. Data are presented as mean ±SEM; n=5. One-way ANOVA with Tukey's post-hoc test.

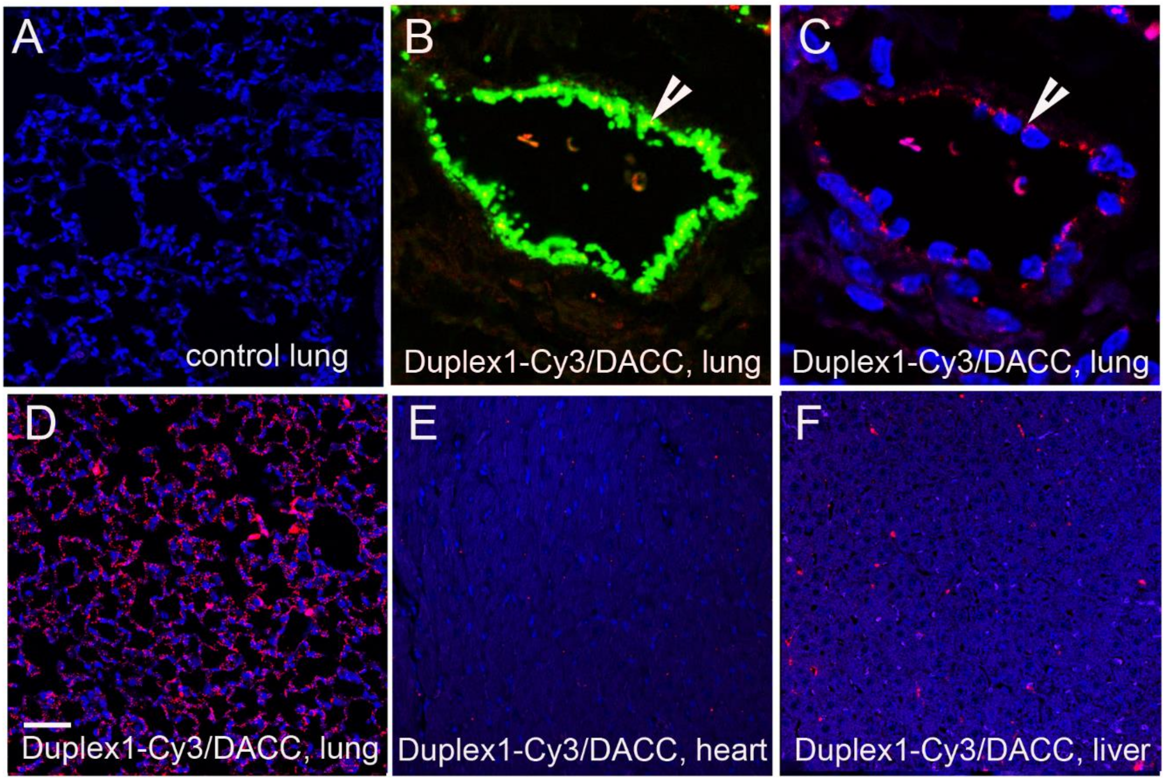


**Online Figure XIII. Distribution of fluorescent siRNA delivered by DACC delivery system in mouse lung, heart and liver**. siRNA-Cy3/DACC (Duplex1-Cy3/DACC) or (vehicle only) were delivered to mice by iv injection and tissue distribution of fluorescent siRNA was studied 24hr later. Representative images of (A) control lung; (B-D) lung (E) heart and (F) liver from mice treated with siRNA-Cy3/DACC. Nuclei are blue (DAPI), siRNACy3 is red and vWF (in B) is green; fluorescent confocal microscopy. (B) and (C) are magnified corresponding images of a lung blood vessel. The arrowheads point to the endothelial layer. In (A, D, E, F) Bar=100 μm and in (B, C) Bar=10 μm.

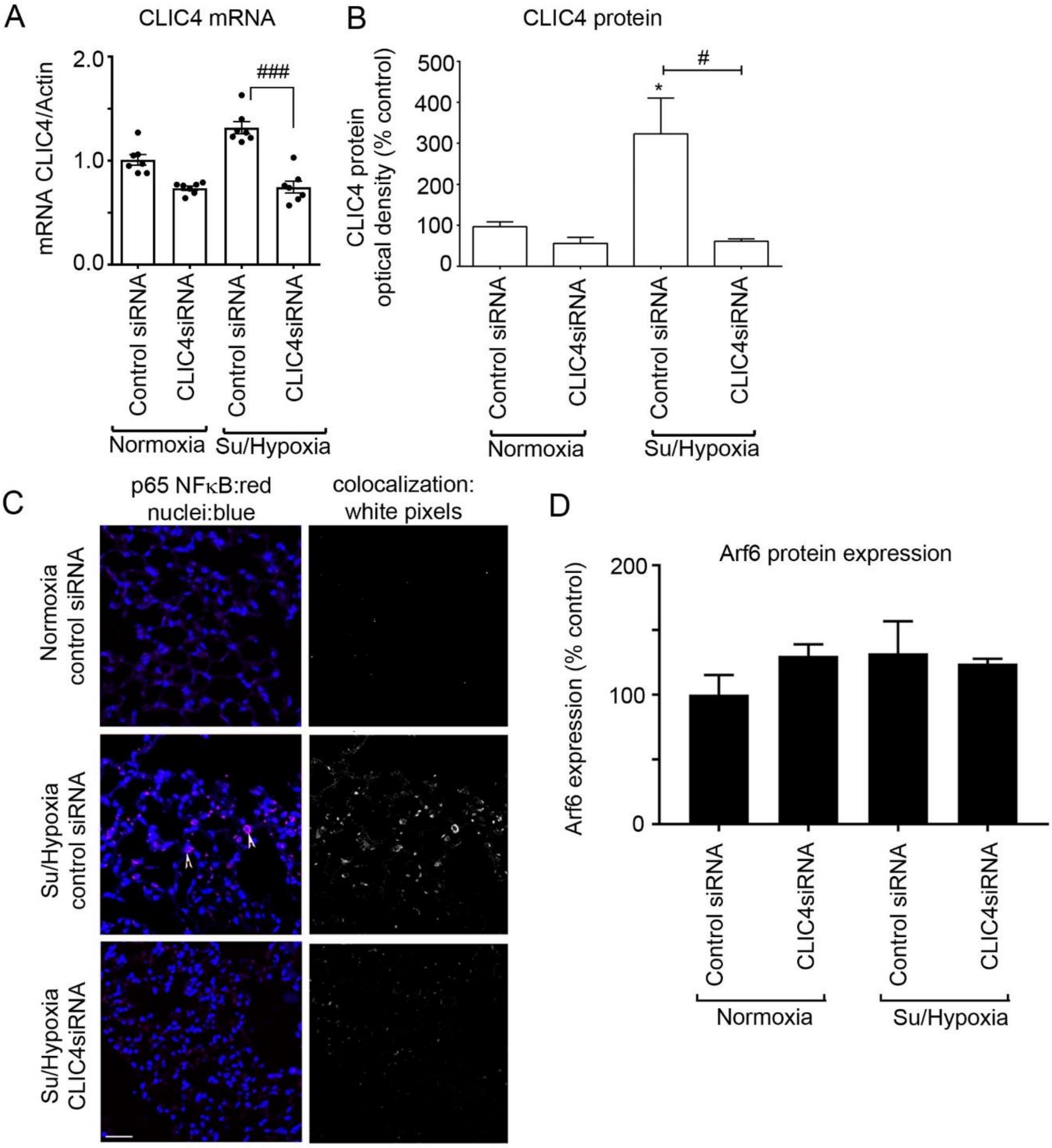


**Online Figure XIV. CLIC4 expression, NFkB nuclear localization and Arf6 expression in lungs of Sugen/hypoxia mice treated with control siRNA or CLIC4 siRNA.** (A) CLIC4 mRNA levels and (B) CLIC4 protein levels in normoxic mice and Sugen/hypoxia mice treated with DACC lipoplex formulated with control siRNA or CLIC4 siRNA formulated with DACC delivery system, as indicated. (C) Delivery of CLIC4 siRNA inhibits nuclear localization of p65NFkB. In confocal images on the left, nuclei are blue (DAPI) and p65NFkB is red. Arrowheads point to nuclear localization of p65NFkB. Corresponding images on the right show colocalizing pixels in white (Image J). Bar=100 µm. (D) Arf6 protein levels in mice treated, as indicated. Data are presented as mean ±SEM; n=8. One-way ANOVA with Tukey's post-hoc test.

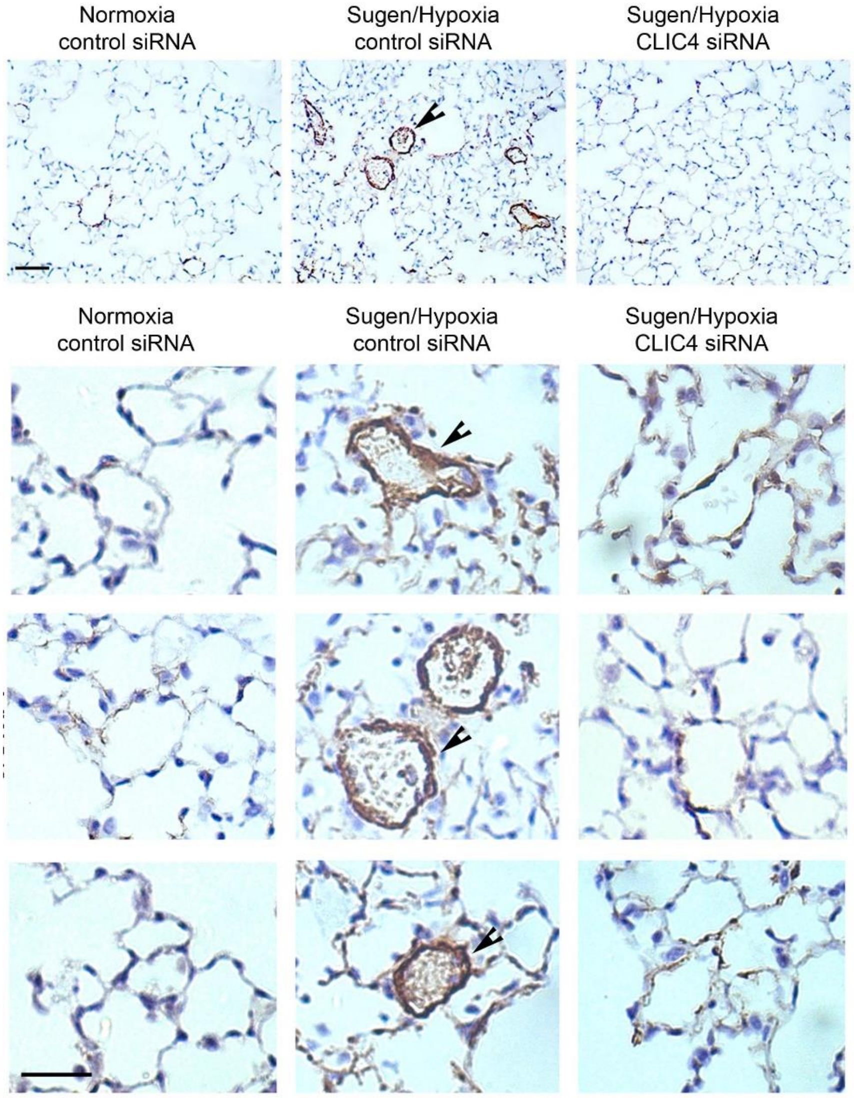


**Online Figure XV. Effects of CLIC4siRNA on muscularization of small intrapulmonary arterioles in Sugen/hypoxia mice.** Images in the top panel and enlarged images in the bottom panel show αSMA staining in mouse lung sections from control mice and Sugen/hypoxia mice treated with non-targeting siRNA (control siRNA). Arrowheads point to remodelled small intrapulmonary vessels. Bar=25 µm. n=7-8.

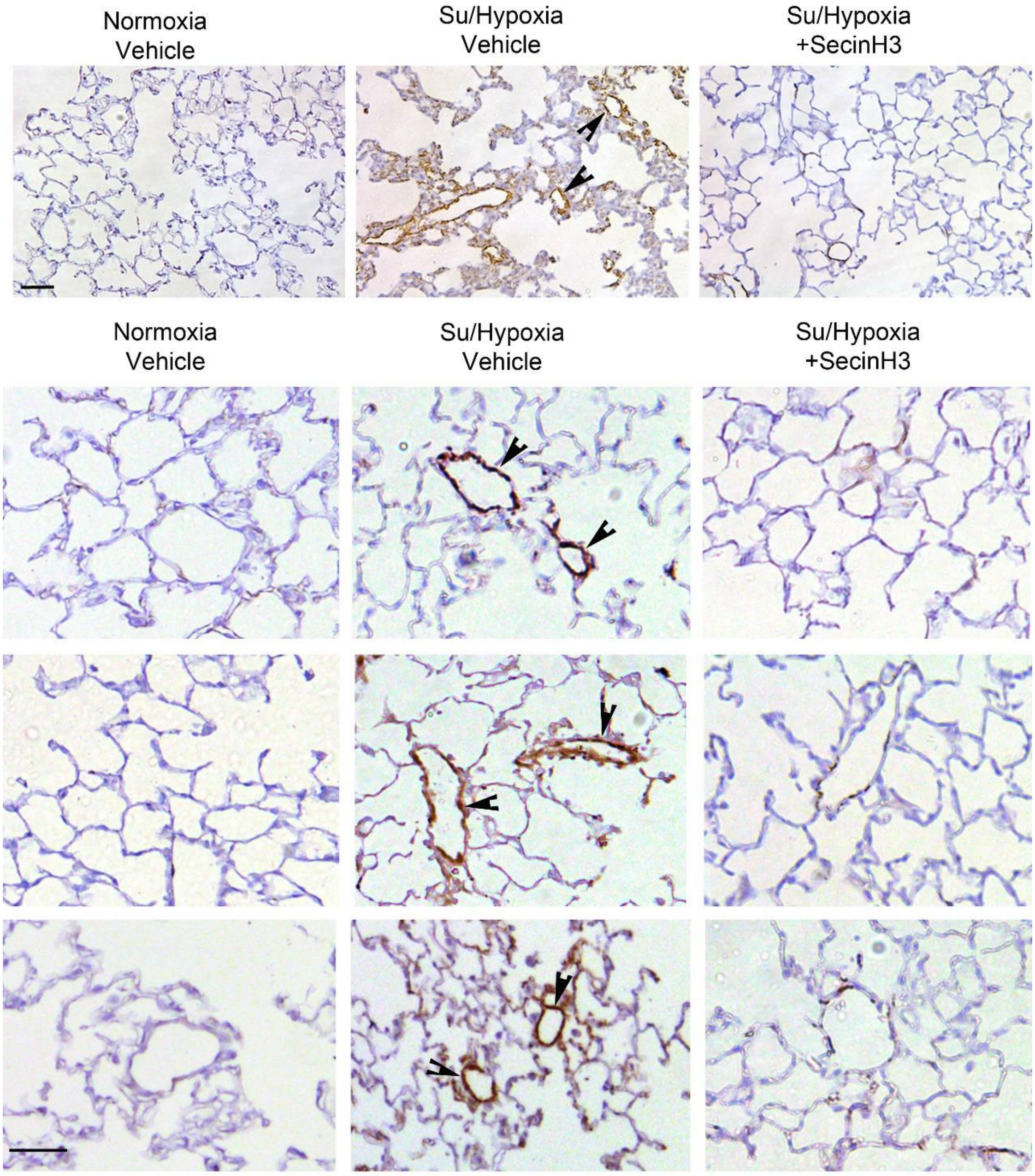


**Online Figure XVI. Effects of SecinH3 on muscularization of small intrapulmonary arterioles in Sugen/hypoxia mice.** Representative images in the top panel and enlarged images in the bottom panel show α-SMA staining in mouse lung sections from control mice (Normoxia Vehicle) and Sugen/hypoxia mice treated with SecinH3 (Su/Hypoxia+SecinH3). Arrowheads point to remodelled small intrapulmonary vessels. Bar=25 µm. n=8.

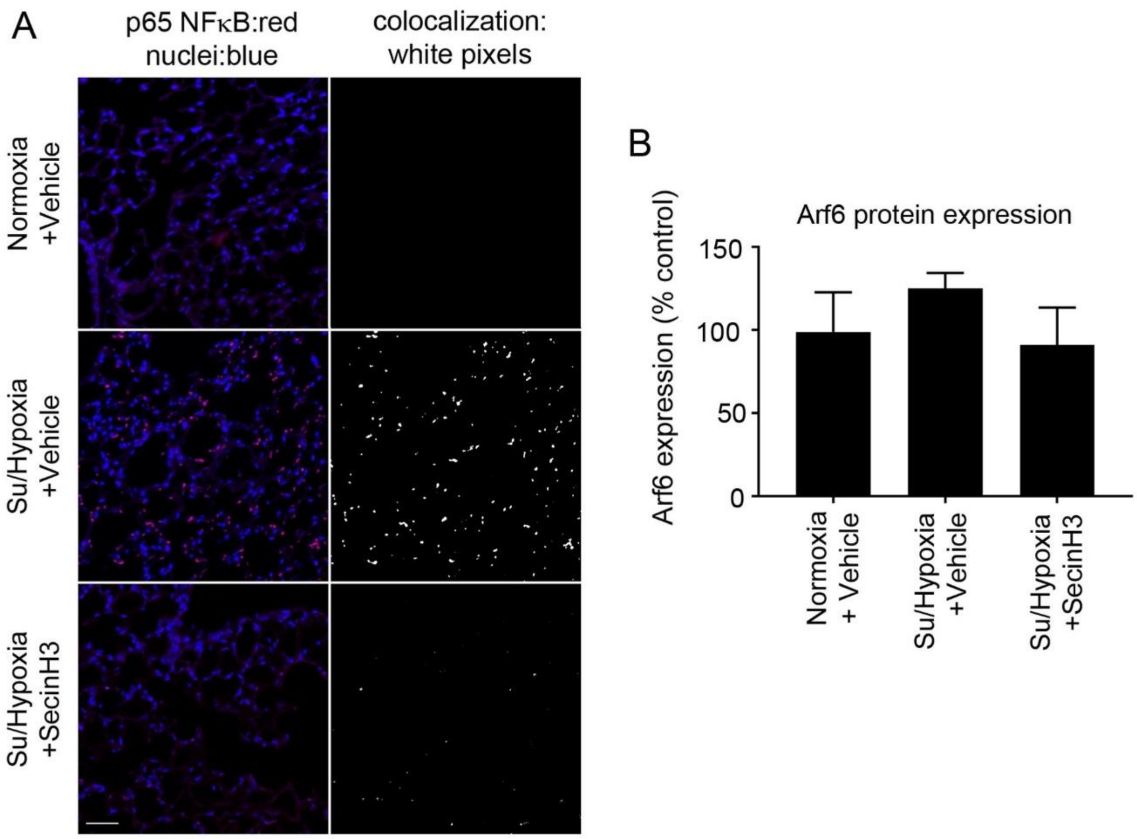


**Online Figure XVII. NFkB nuclear localization and Arf6 expression in lungs of Sugen/hypoxia mice treated with SecinH3.** (A) SecinH3 inhibits nuclear localization of p65NFkB. In confocal images on the left, nuclei are blue (DAPI) and p65NFkB is red. Corresponding images on the right show colocalizing pixels in white (Image J). Bar=100 µm. (B) Arf6 protein levels in mice treated, as indicated; n=8.

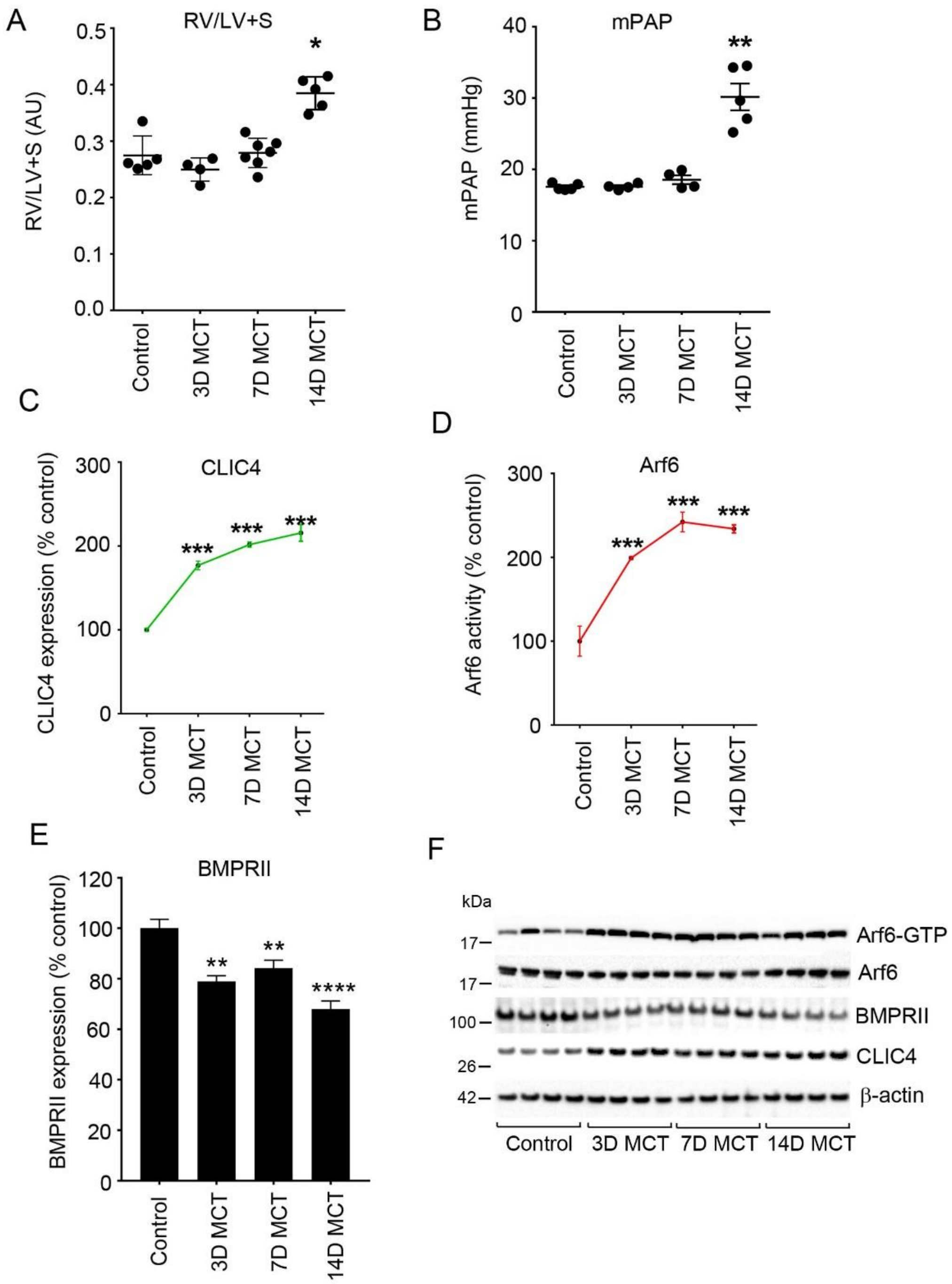


**Online Figure XVIII. Time course of changes in CLIC4 expression, Arf6 activation and BMPRII expression in MCT rats**. (A) Right ventricular hypertrophy indicated by RV/LV+S; (B) Mean artery pressure (mPAP); (C-F) graphs and representative western blots showing Arf6 and Arf1 activity, along with CLIC4 and BMPRII expression in the lungs of control rats and rats treated with MCT for 3, 7 and 14 days. Data are expressed as mean ±SEM; n = 4-6, one-way ANOVA with Tukey's post-hoc test. *P<0.05, **P<0.01, ***P<0.001, compared to controls.

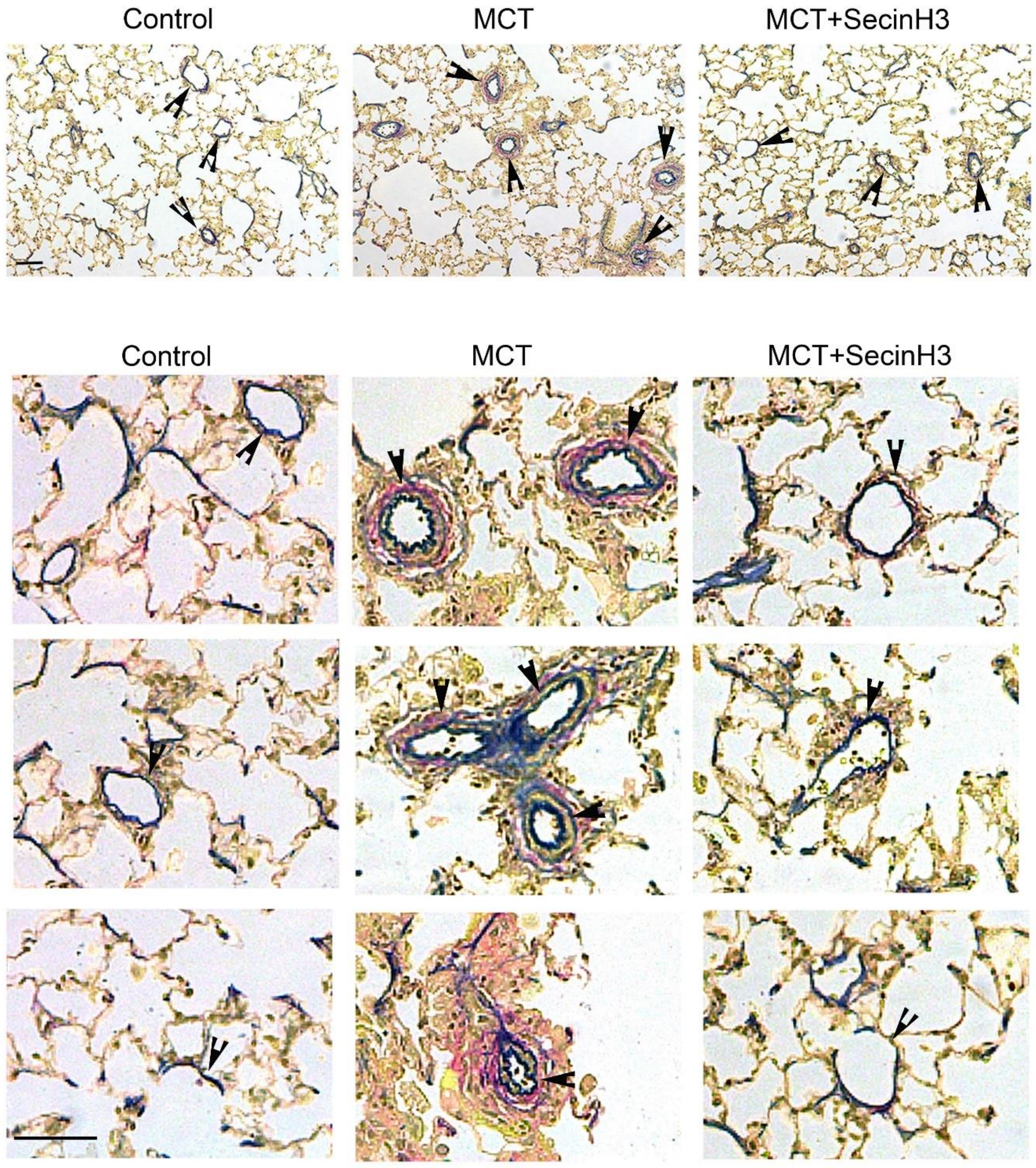


**Online Figure XIX. Effects of SecinH3 on remodelling of small intrapulmonary arterioles in MCT rats**. Representative images in the top panel and enlarged images in the bottom panel show Elastic van Gieson (EVG) staining of fully muscularized peripheral arteries with double elastic lamina in MCT rat lung and single elastic laminae in control and SecinH3-treated rat lung (arrowheads). Bar=25 µm. n=6.

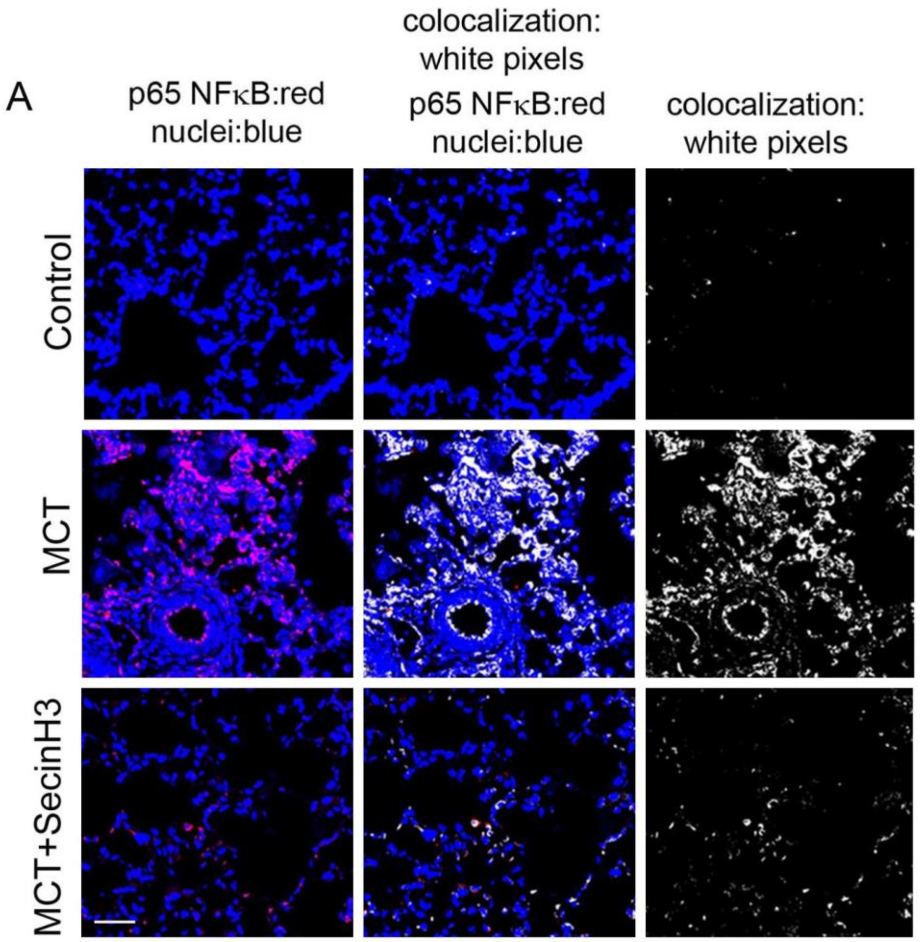


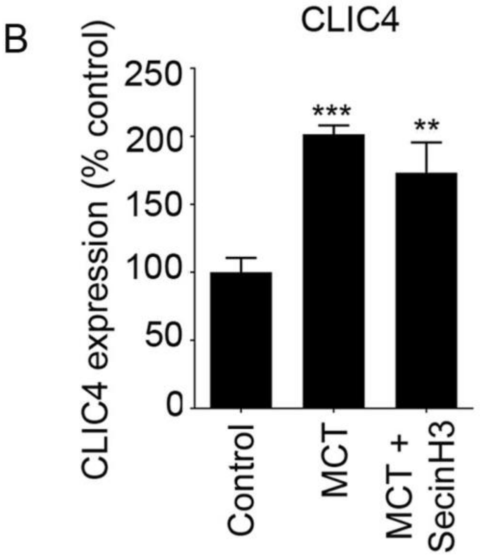


**Online Figure XX. NFκB nuclear localization and CLIC4 expression in lungs of MCT rats.** (A) SecinH3 inhibits nuclear localization of p65NFkB. In confocal images on the left, nuclei are blue (DAPI) and p65 NFκB is red. Corresponding images on the right show colocalizing pixels in white (Image J). Bar=50 µm. (B) CLIC4 protein levels in rats treated, as indicated. Data are expressed as mean±SEM, n=6. **P <0.01, ***P<0.001 compared to control. One-way ANOVA with Tukey's post-hoc test.

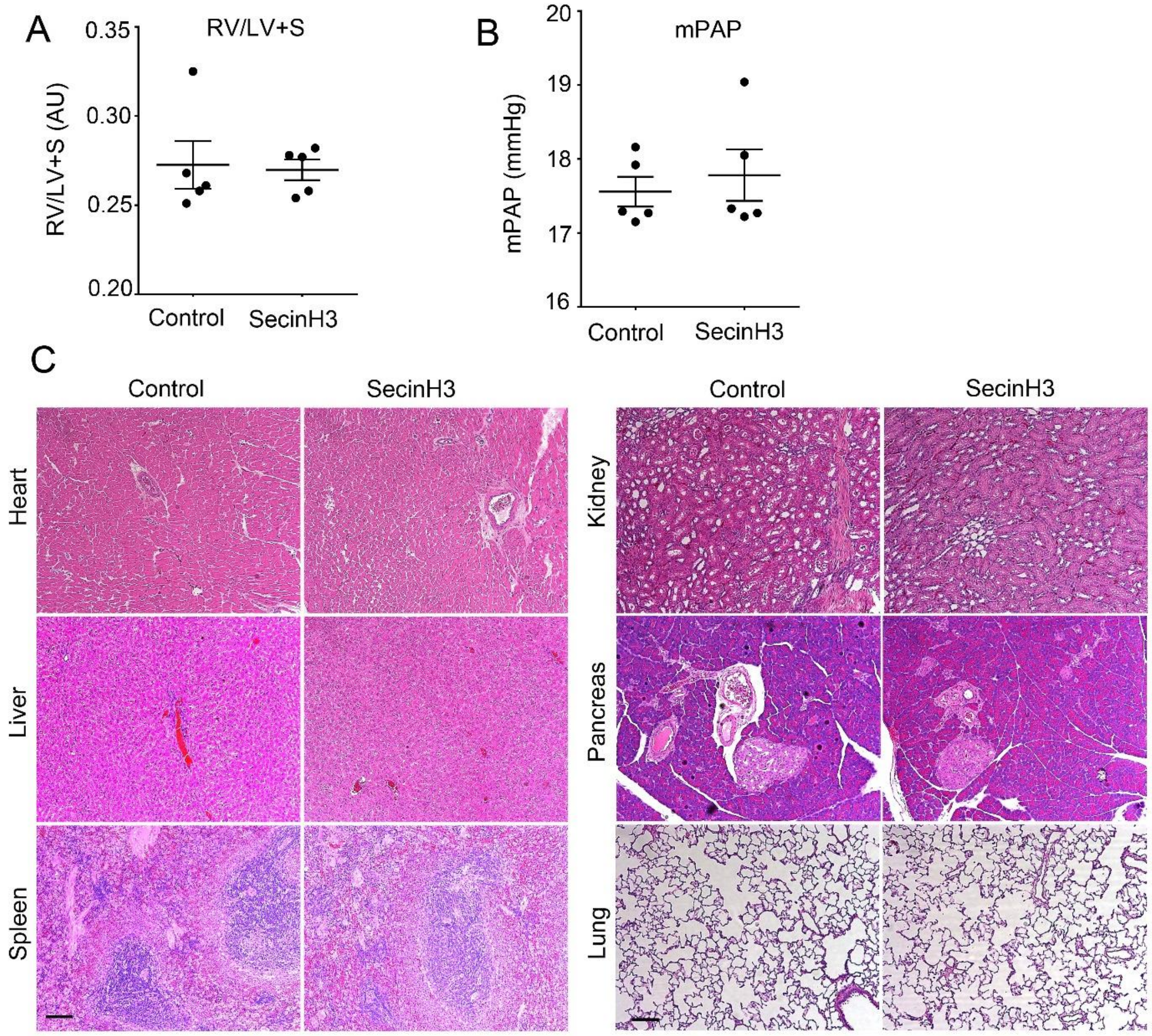


**Online Figure XXI. SecinH3 treatment does not alter mean pulmonary artery pressure, right heart size or the histopathology of major organs in rats.** (A) Mean artery pressure (mPAP); (B) RV/LV+S; and (C) haematoxylin-eosin staining of tissues from the heart, liver, spleen, kidney, pancreas and lung from untreated rats and rats treated with SecinH3 (2.5 mg/kg body weight, 14 days). Bar=50 µm. Data are expressed as mean ±SEM, n=5; Mann-Whitney U test.
